# Nuclear importin α1 AtIMPA1 regulates stomatal reopening to defend against *Pst* DC3000 by orchestrating ABA signaling

**DOI:** 10.1111/tpj.71175

**Published:** 2026-10-29

**Authors:** Ting Zhang, Kang Wang, Yi Guan, Jingao Zhang, Charles Tetteh, Chenxi Liu, Cheng Zhang, Pinyuan Jin, Sheng Luo, Yiling Wang, Huajian Zhang

**Affiliations:** ^1^ Key Laboratory of Agri‐products Quality and Biosafety (Ministry of Education) Anhui Agricultural University Hefei 230036 China; ^2^ Anhui Province Key Laboratory of Crop Integrated Pest Management, Department of Plant Pathology, College of Plant Protection Anhui Agricultural University Hefei 230036 China

**Keywords:** importin α1, *Pst* DC3000, COR, stomatal reopening, ABA signaling

## Abstract

Importin α family proteins serve as essential carriers for transporting specific disease‐resistant proteins into the nucleus, enabling these proteins to function properly. However, whether importin α proteins are involved in plant immunity remains unclear. Here, we characterized a guard cell‐expressed protein importin α1 AtIMPA1, which contributed to resistance against *Pseudomonas syringae* pv. *tomato* DC3000 (*Pst* DC3000). When dip‐inoculated with *Pst* DC3000, the *impa1* mutants exhibited increased susceptibility, along with significant stomatal reopening induced by the phytotoxin coronatine (COR) secreted by *Pst* DC3000; while overexpression of *AtIMPA1* enhanced resistance to *Pst* DC3000 with compromised stomatal reopening induced by COR. Notably, following syringe‐infiltration of *Pst* DC3000, the *impa1* mutants showed increased resistance, whereas overexpression lines were more susceptible to *Pst* DC3000, indicating that COR‐triggered stomatal reopening is a primary determinant in AtIMPA1‐mediated disease resistance. Further investigations revealed that AtIMPA1 regulated COR‐triggered stomatal reopening is linked to abscisic acid (ABA) signaling. Mutation of *AtIMPA1* attenuated the ABA‐mediated inhibition of root length and seed germination, and disrupted the ABA‐induced protein abundance and localization of the ABA‐responsive protein, supporting its role in regulating the ABA response. These findings shed light on the functional significance of AtIMPA1 in COR‐triggered stomatal reopening and provided new insights into how importin α proteins orchestrate ABA signaling to regulate plant immunity.

## Significance Statement

This study reveals that the guard cell‐expressed importin α protein AtIMPA1 modulates coronatine‐triggered stomatal reopening through abscisic acid signaling to restrict *Pst* DC3000 entry. These findings provide novel insights into how plants integrate pathogen‐derived signals with hormonal responses to defend against pathogen infection.

## INTRODUCTION

Bacterial infections in plants typically begin with the colonization of the plant surface, followed by entry through wounds and stomata, multiplication within the apoplastic space of the affected tissues, and dissemination of bacteria to neighboring plants (Hou et al., 2024; Zeng et al., 2011). The regulation of stomatal opening and closure represents an evolutionary battle between plants and pathogens, characterized by adaptive strategies that impact both plant resistance and pathogen virulence (Hou et al., 2024; Melotto et al., 2017). When plants detect the presence of bacterial pathogens, they initiate signaling pathways that result in the closure of the stomata, thereby lessening the likelihood of pathogen entry into the leaf interior, a process known as stomatal immunity. Pattern recognition receptors (PRRs) are known to trigger stomatal closure after recognizing conserved pathogen‐associated molecular patterns (PAMPs), which include bacterial flagellin (a flagellin peptide, flg22) (Fan et al., 2004; Hou et al., 2024; Melotto et al., 2006). As a counter‐defense mechanism, certain pathogens, including *Pseudomonas syringae* pv. *tomato* DC3000 (*Pst* DC3000), have also evolved the phytotoxin coronatine (COR) to antagonize plant stomatal closure during the pre‐invasive stage (Liu et al., 2024). COR mimics jasmonoyl‐isoleucine (JA‐Ile), the active form of JA, and activates JA signaling by targeting the JA receptor complex (COI1‐JAZ) and then modulates the expression of three transcription factors (TFs) of the NAC family, ANAC019, ANAC055, and ANAC072, via the master transcriptional factor MYC2 (Bu et al., 2008; Zheng et al., 2012). Upon successful passage through the stomata and entering the apoplastic region of leaves, plants initiate a series of defense signaling pathways. This includes the activation of mitogen‐activated protein kinases (MAPKs), calcium influx, and the generation of reactive oxygen species (ROS), a process referred to as apoplastic immunity (Su et al., 2017; Zeng et al., 2011).

Abscisic acid (ABA) is a crucial phytohormone that orchestrates multiple physiological processes in plants, including stomatal closure, seed germination, inhibition of root growth, and responses to both biotic and abiotic stresses (Chen et al., 2020). The ABA‐signaling pathway commences with ABA recognition by the PYR1/PYL/RCAR receptor proteins, followed by interaction with and inactivation of clade‐A protein phosphatase type 2Cs (PP2Cs), such as ABI1, ABI2, HAB1, HAB2, and PP2CA/AHG3, thereby relieving their inhibition on three ABA‐activated SNF1‐related protein kinases (SnRK2s): SnRK2.2/D, SnRK2.3/I, and SnRK2.6/E/OST1 (Belda‐Palazon et al., 2019; Gonzalez‐Guzman et al., 2012; Waterland et al., 2010). ABA serves as a pivotal signaling molecule in guard cells, contributing to plant resistance against bacterial infections by promoting stomatal immunity during the pre‐invasion phase. For instance, the ABA‐deficient mutant, *aba3‐1*, and the ABA‐signaling mutant, *ost1‐2*, do not close their stomata in response to *Pst* DC3000 or PAMPs (Melotto et al., 2006). Mutation of *OST1* resulted in insensitivity to ABA‐triggered stomatal closure and ABA inhibition of light‐induced stomatal opening (Mine et al., 2017; Wang et al., 2023). In guard cells, COR inhibits the ABA signal transduction pathway and induces reopening of closed stomata, allowing the pathogen to enter the plant's apoplast (He et al., 2012). COR also promotes the accumulation of ABI1, which inhibits the activity of SnRK2 kinases, ultimately leading to the opening of the stomata (Liu et al., 2024). These findings underscore the involvement of the ABA pathway in regulating stomatal immunity.

Numerous proteins, including TFs, splicing factors, and ribosomal subunit proteins, which contain nuclear localization signals (NLS) or nuclear export signals (NES), are conventionally transported between the nuclear and cytoplasmic compartments by karyopherins (Kim et al., 2017; Yang, Guo, et al., 2023). Among those karyopherins, the importin α and β families are the most important ones. Classical NLSs (cNLSs) of cargo proteins are recognized by the adaptor protein importin α, which interacts with the carrier protein importin β to facilitate the nuclear import of cargo proteins through nuclear pore complexes (NPCs) (Köhler et al., 1999; Wang, Zhang, et al., 2024; Yang, Guo, et al., 2023). The small GTPase Ran, along with accessory factors, subsequently disassembles the ternary complex and facilitates the release of the cargo protein within the nucleus (Wang, Zhang, et al., 2024; Yang, Guo, et al., 2023). These canonical or cNLSs have the consensus sequences [K(K/R)X(K/R)] and [(K/R)(K/R)X_10‐12_(K/R)_3/5_], respectively (Ludke et al., 2021). It has been reported that importin α proteins contribute to various cellular processes in plants, including growth and development, hormonal signaling, and responses to environmental changes (Bhattacharjee et al., 2008; Liu, Xiong, et al., 2019; Ludke et al., 2021). Importin α1 is a member of the importin α family. In human cells, importin α1 (KPNA1) is a mediator of nucleocytoplasmic transport and participates in the nuclear import process of nuclear receptor or other regulatory proteins that modulate neuronal differentiation, synaptic function, and cancer cell variation (Pasha et al., 2021; Zhao et al., 2023). In rice, importin α1 (IMPα1b) serves as a nuclear transport receptor to facilitate the nuclear import of T3 effectors, PthXo1 and TALI, from *Xanthomonas oryzae* pv. *Oryzae* (*Xoo*) and *Xanthomonas oryzae* pv. *oryzicola* (*Xoc*), respectively (Peng et al., 2022). Although in *Arabidopsis thaliana*, importin α1 (AtIMPA1) was reported to interact with importin β1 (AtKPNB1), which is required for ABA response and drought tolerance (Luo et al., 2013); the function of AtIMPA1 remains unclear, particularly regarding its roles in plant immunity. In this study, we found that the importin α1 protein, AtIMPA1, positively modulates resistance to *Pst* DC3000 through COR‐triggered stomatal reopening linked to ABA. Our findings highlight the positive regulatory effect of Arabidopsis AtIMPA1 on resistance to *Pst* DC3000 through COR‐triggered stomatal reopening by the orchestration of ABA signaling.

## RESULTS

### Mutation of AtIMPA1 reduces resistance to *Pst* DC3000 by dipping inoculation

The genome of *A. thaliana* Col‐0 encodes nine importin α isoforms (Ludke et al., 2021). Based on the Plant eFP Browser database (https://bbc.botany.utoronto.ca/efp/cgi‐bin/efpWeb.cgi) and a screening of plant disease symptoms in Arabidopsis T‐DNA insertion mutants of the importin α genes upon *Pst* DC3000 inoculation, an AtIMPA1 mutant (SALK_105557, *impa1‐1* mutant) was identified due to its increased susceptibility to *Pst* DC3000. AtIMPA1 (Importin α 1, AT3G06720) encodes a protein consisting of 532 amino acids that features an N‐terminal auto‐inhibitory importin‐β‐binding (IBB) domain followed by a series of 10 armadillo (ARM) repeats that form the NLS‐binding cleft (Figure S1A,B). AtIMPA1 shares 52.7% homology with human KPNA1 (Figure S2A). Phylogenetic analysis showed that importin α1 proteins from *Vitis vinifera*, *Ricinus communis*, *Populus trichocarpa*, and *A. thaliana* clustered together in a distinct branch (Figure S2B), suggesting a potential shared ancestry among these species.

In addition to the *impa1‐1* mutant, another independent T‐DNA insertion mutant line (SALK_082616, *impa1‐2* mutant) with reduced *AtIMPA1* expression compared with Col‐0 was isolated (Figure S1A,C,D). The 35S‐*AtIMPA1*‐GFP construct was introduced into the Col‐0, resulting in the generation of four overexpression lines (OE‐1#, OE‐2#, OE‐6#, and OE‐11#). Since the OE‐6# (OE1) and OE‐11# (OE2) lines exhibited higher transcript and protein levels than the other lines (Figure S1A,D,E), they were selected for further analysis. Two complementation lines, CL‐1# and CL‐2#, were generated by transforming the *AtIMPA1pro*‐GFP construct into the *impa1‐1* and *impa1‐2* mutants, restoring *AtIMPA1* expression levels comparable to those in Col‐0 (Figure S1F,G). Next, all genotypes were dip‐inoculated with virulent hemibiotrophic bacteria *Pst* DC3000. At 3 days post‐inoculation (dpi), the *impa1* mutants displayed more pronounced yellowing and wilting, along with higher bacterial titers than Col‐0, while overexpression lines exhibited increased resistance to *Pst* DC3000 (Figure 1A). The complementation lines showed disease symptoms and bacterial titers similar to those in Col‐0 (Figure S3A–C). Upon *Pst* DC3000 infection, the expression level of *AtIMPA1* was significantly induced, and the transcript levels of defense‐related genes *PR1* and *NPR1* were markedly reduced in the *impa1* mutant but enhanced in *AtIMPA1*‐overexpression lines compared with Col‐0 (Figure 1B–D). Collectively, these findings indicate that AtIMPA1 positively modulates resistance to *Pst* DC3000 by modulating the expression of defense‐related genes.

**Figure 1:**
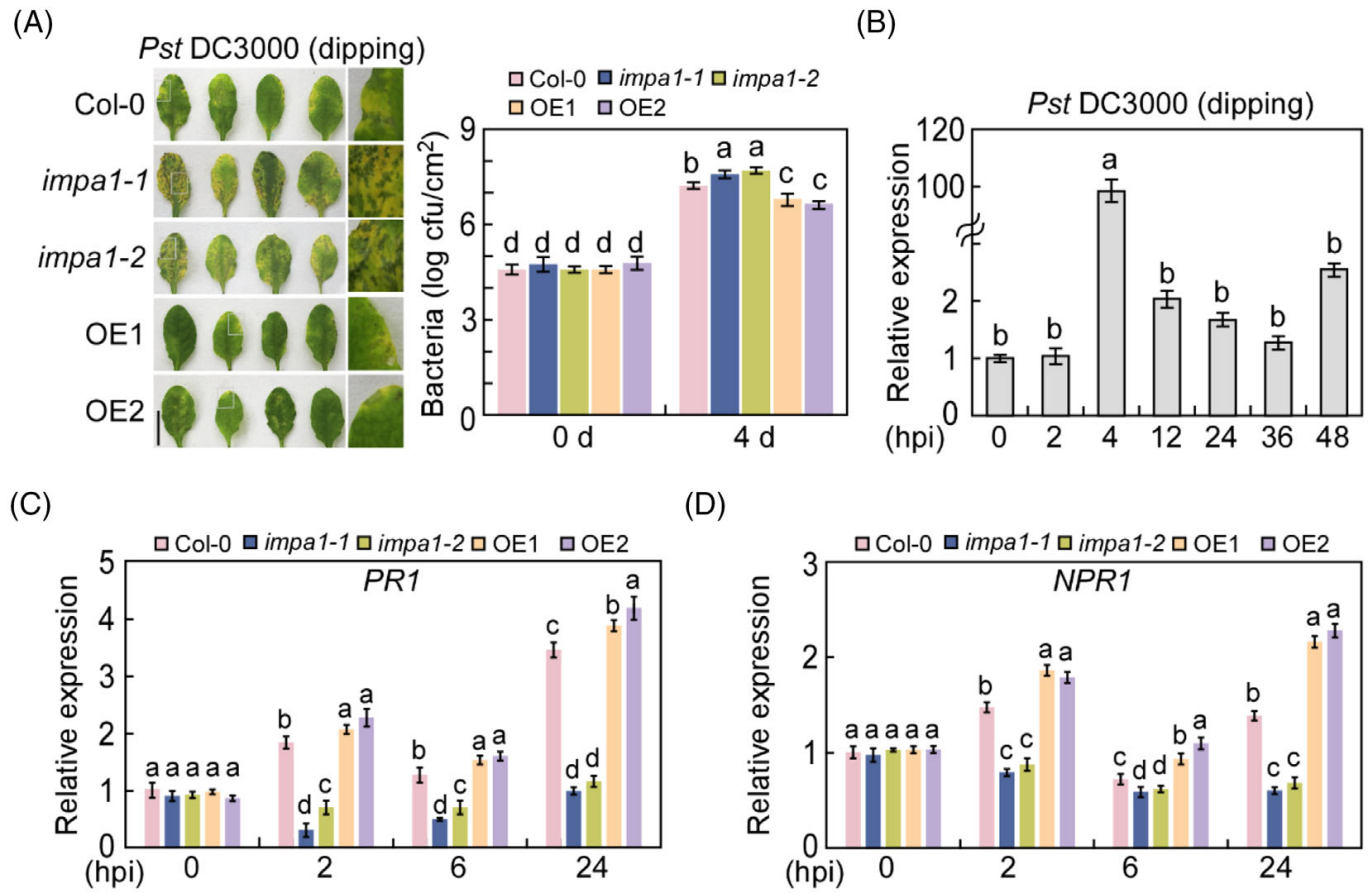
AtIMPA1 plays a positive role in resistance to *Pst* DC3000. (A) The disease symptoms and titers of 5‐week‐old Col‐0, *impa1‐1*, *impa1‐2* mutant, OE1, and OE2 plants were evaluated after dip‐inoculated with *Pst* DC3000 (1 × 10^7^ cfu/mL) and photographed at 3 days post‐inoculation (dpi). Error bars of bacterial titers analysis are SDs of three biological replicates. Statistical analysis was performed by one‐way ANOVA with Tukey HSD using GraphPad Prism 8.0.1, and letters represents significant differences at *P* < 0.05. Scale bar = 1 cm. (B) Reverse transcriptase‐quantitative polymerase chain reaction (RT‐qPCR) analyses of the expression levels of *AtIMPA1* gene in Col‐0 at different time after dip‐inoculated with *Pst* DC3000 (1 × 10^7^ cfu/mL). Error bars are SDs of three biological replicates. Statistical analysis was performed by one‐way ANOVA with Tukey HSD using GraphPad Prism 8.0.1, and letters represents significant differences at *P* < 0.01. (C, D) RT‐qPCR analyses of the expression levels of the *PR1* and *NPR1* gene in Col‐0, *impa1* mutants and OE plants at 0, 2, 6, and 24 h after dip‐inoculated with *Pst* DC3000 (1 × 10^7^ cfu/mL). Error bars are SDs of three biological replicates. Statistical analysis was performed by one‐way ANOVA with Tukey HSD using GraphPad Prism 8.0.1, and letters represents significant differences at *P* < 0.05.

### AtIMPA1 mediates the import of proteins containing cNLS and is expressed in guard cells

cNLSs are short peptides that mediate the transport of proteins from the cytoplasm into the nucleus, and the localization of cNLS in Col‐0 and *impa1* mutant expressing a cNLS‐RFP (cNLS fused with RFP) was examined. The cNLS‐RFP was predominantly localized in the nucleus of Col‐0 (94% ± 2%), while it exhibited reduced nuclear localization in the *impa1‐1* mutant (28% ± 6%). The fluorescence intensity in Col‐0 displayed distinct primary peaks, whereas the *impa1‐1* mutant exhibited erratic peak patterns (Figure 2A). These observations suggest that AtIMPA1 facilitates the transport of cNLS‐containing proteins into the nucleus, which is a key characteristic of importin α proteins. Then, a stable transgenic Arabidopsis line expressing AtIMPA1‐GFP driven by the native promoter was generated to assess its expression pattern. Punctate GFP signals were observed in various tissues, including the roots, flowers, and leaves. Interestingly, GFP signals were most pronounced in the guard cells of the epidermis at the cellular level (Figure 2B). Next, the subcellular localization of co‐expressing AtIMPA1‐GFP and cNLS‐RFP was examined in *Arabidopsis* mesophyll protoplasts. The fluorescence signals of AtIMPA1‐GFP were completely co‐localized with cNLS‐RFP in the nucleus (Figure 2C). This was further supported in *Nicotiana benthamiana*, where GFP fluorescence overlapped with DAPI staining (Figure 2D). Collectively, these results suggest that the nucleus‐localized protein AtIMPA1 mediates the import of proteins containing cNLS and is expressed in various tissues, including guard cells.

**Figure 2:**
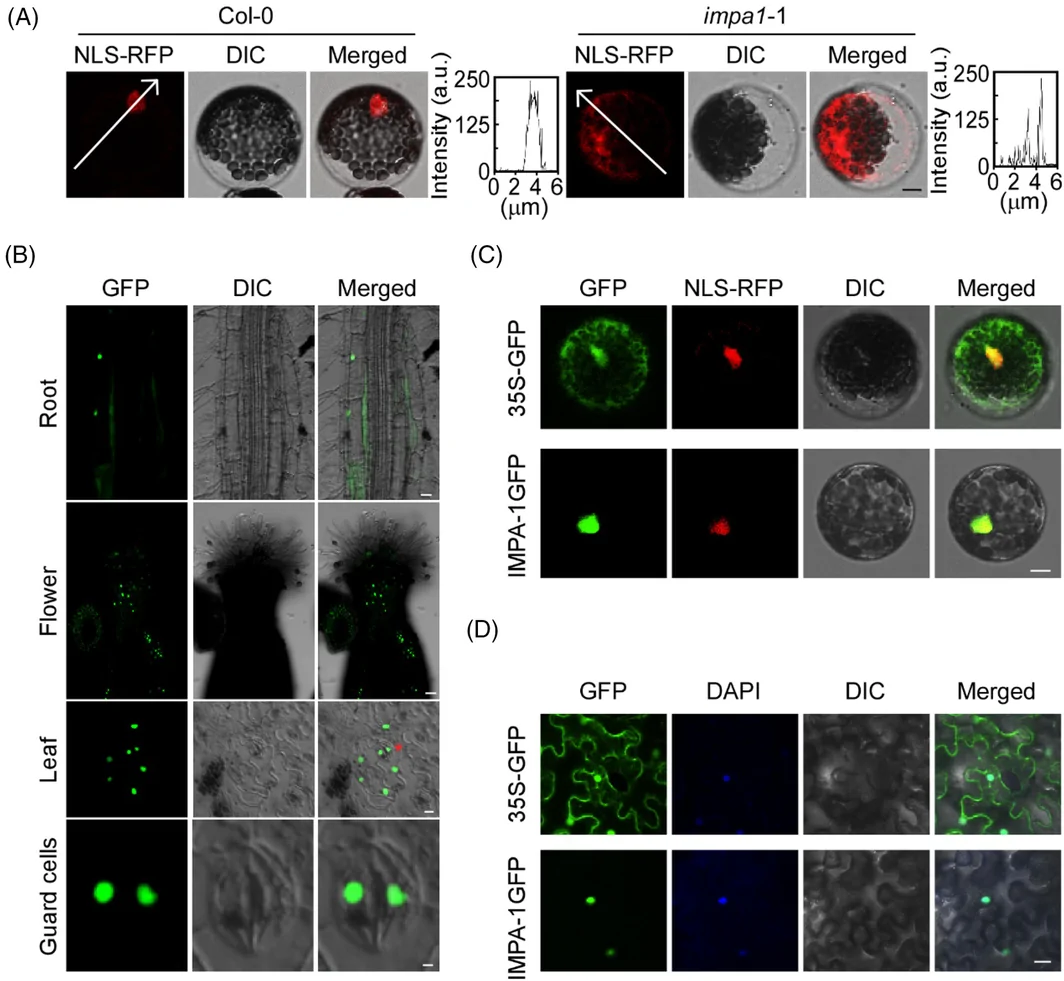
AtIMPA1 mediates the import of proteins containing classical nuclear localization signals (cNLS) and is expressed in guard cells. (A) Localization of cNLS‐RFP in Col‐0 and *impa1‐1* mutant mesophyll protoplasts. Insets highlight areas analyzed by line scan. The white line represents cNLS‐RFP. Scale bar = 50 μm. (B) Expression of Arabidopsis transgenic seedlings with the AtIMPA1‐GFP driven by the native promoter. Scale bar (Root/Flower/Leaf/Guard cells) = 200/100/100/10 μm. The red asterisk represents a smaller version of the guard cells. (C) Localization of AtIMPA1‐GFP in Col‐0 mesophyll protoplasts. *p*UC19‐GFP was used as a negative control. Scale bar = 50 μm. (D) Localization of AtIMPA1‐GFP in *Nicotiana benthamiana*. *p*BINPLUS‐GFP was used as a negative control. Scale bar = 50 μm.

### Overexpression of AtIMPA1 inhibits COR‐triggered stomatal reopening

Upon pathogen perception, stomata rapidly close within 1.5 h to restrict pathogen entry; however, as a counter‐defense mechanism, *Pst* DC3000 has evolved the COR to antagonize this immune response, thereby triggering stomatal reopening 4 h post‐infection. Given that *AtIMPA1* is expressed in guard cells, a stomatal aperture assay was employed to test whether its resistance to *Pst* DC3000 was associated with stomatal movement. Stomata of overexpression lines were closed similarly to those observed in Col‐0 at 1.5 h after *Pst* DC3000 treatment, while the stomata of *impa1* mutants did not show complete closure until 2 h after *Pst* DC3000 treatment (Figure 3A,B). The PAMP flg22, derived from *Pst* DC3000, induced plant stomatal closure to restrict bacterial entry after *Pst* DC3000 infection (Chan et al., 2020; Melotto et al., 2006). Consistent with the observations from plants treated with *Pst* DC3000, stomatal closure upon flg22 treatment was delayed in *impa1* mutants (Figure 3A,B). These findings suggest that although the mutation of *AtIMPA1* leads to a slight delay in bacterium‐ or flg22‐induced stomatal closure, it does not affect ultimate stomatal closure. *Pst* DC3000 has evolved the phytotoxin COR to facilitate the reopening of stomata, thereby enabling bacterial entry within 4 hpi (Guo et al., 2018; Melotto et al., 2006). A COR‐deficient mutant (*Pst* DC3000 COR^−^, *Pst* ^−^) strain was used to further assess the potential effect of AtIMPA1 in COR‐triggered stomatal reopening. The stomata of Col‐0 and the *impa1* mutants remained closed within 4 h after *Pst* ^−^ treatment, while their stomata prominently reopened at 4 h after *Pst* DC3000 and *Pst* ^−^ accompanied by COR treatment, with the *impa1* mutants exhibiting significant stomatal openness than the Col‐0 (Figure 3C,D). Conversely, the stomata of overexpression lines consistently remained closed after treatment with *Pst* DC3000, *Pst* ^−^, and *Pst* ^−^ accompanied by COR (Figure 3C,D). The stomata of complementation lines were closed at 2 h after incubation with *Pst* DC3000 and exhibited stomatal reopening at 4 h when exposed to *Pst* DC3000, COR, or a *Pst* ^−^ strain accompanied by COR treatment, consistent with Col‐0 (Figure S3D). These findings indicate that lines overexpressing *AtIMPA1* were compromised in COR‐triggered stomatal reopening, thereby contributing to resistance against *Pst* DC3000.

**Figure 3:**
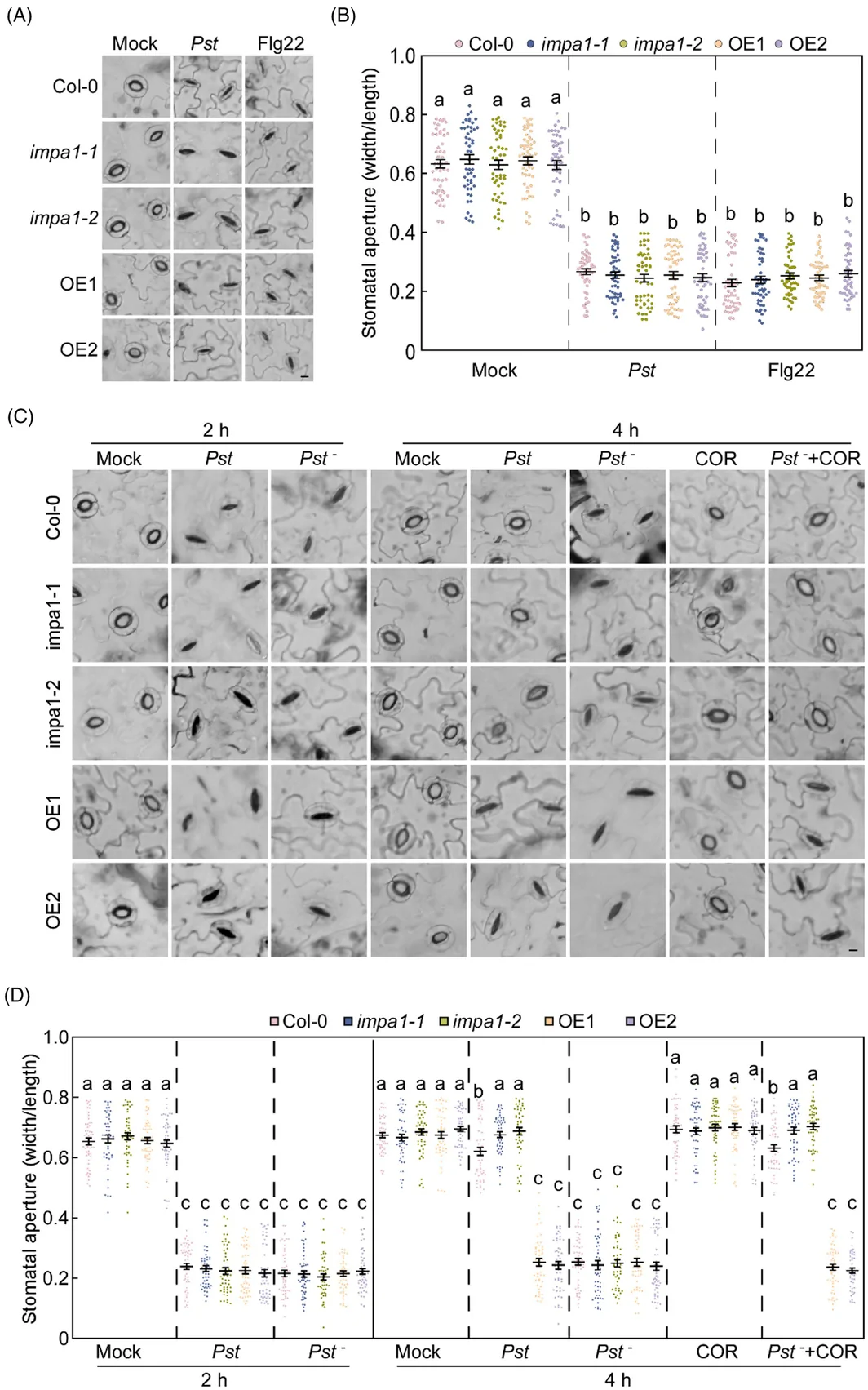
Overexpression of *AtIMPA1* inhibits coronatine (COR)‐triggered stomatal reopening. (A, B) The morphology and aperture of stomata were examined and statistically analyzed in Col‐0, *impa1‐1*, *impa1‐2* mutant, OE1, and OE2 plants at 2 h after *Pst* DC3000 and flg22 treatment. Data are representative of three independent experiments with similar results. Quantitative analysis of stomatal aperture was performed from 50 stomata fields using ImageJ. Values represent average ± SEM. Statistical analysis was performed by one‐way ANOVA with Tukey HSD using GraphPad Prism 8.0.1, and letters represent significant differences at *P* < 0.01. (C, D) The morphology and aperture of stomata were examined and statistically analyzed in Col‐0, *impa1‐1*, *impa1‐2* mutant, OE1, and OE2 plants at 2 and 4 h after *Pst* DC3000, *Pst* ^−^, COR, and *Pst* ^−^ accompanied by COR treatment. Data are representative of three independent experiments with similar results. Quantitative analysis of stomatal aperture was performed from 50 stomata fields using ImageJ. Values represent average ± SEM of 50 stomata. Statistical analysis was performed by one‐way ANOVA with Tukey HSD using GraphPad Prism 8.0.1, and letters represent significant differences at *P* < 0.05.

### Mutation of AtIMPA1 enhanced resistance to *Pst* DC3000 by syringe‐inoculation

Compared with dipping inoculation with *Pst* DC3000, syringe‐infiltration can bypass stomatal immunity and allow for infection through apoplastic defenses. To further investigate whether the higher susceptibility of the *impa1* mutants observed in the dip‐inoculation assay was attributable to apoplastic defense, all genotypes were syringe‐infiltrated with *Pst* DC3000. In contrast to the results obtained from dipping inoculation, the overexpression lines displayed more pronounced symptoms of bacterial infection, including increased yellowing and wilting, as well as elevated bacterial titers compared with Col‐0 at 2 days post‐syringe‐infiltration. Meanwhile, the *impa1* mutants showed enhanced resistance to *Pst* DC3000 (Figure 4A). COR has been reported to be largely dispensable for bacterial multiplication once pathogens bypass the stomata and are directly infiltrated into the apoplast (Melotto et al., 2006). To investigate which *Pst* DC3000 virulence factors are involved in suppressing apoplastic defense in an AtIMPA1‐dependent manner, the non‐pathogenic *hrcC* ^−^ mutant, a well‐characterized strain defective in the secretion of T3SS‐dependent effectors, was employed. The *hrcC* ^−^ mutant grew very poorly in Col‐0 and mutants when infiltrated into the apoplast (Figure 4A), suggesting that the contrasting apoplastic phenotypes observed with virulent *Pst* DC3000 require T3 effector‐mediated suppression of immunity, in which AtIMPA1 functions as a negative regulator. Since stomatal closure is only one of several functional outputs induced by PAMPs (Zeng et al., 2011), a possible effect of AtIMPA1 on the flg22‐triggered apoplastic defense was evaluated. Before inoculation with *Pst* DC3000, all genotypes were pretreated with 100 nM flg22 or water (mock treatment) for 24 h. The *impa1* mutants exhibited reduced bacterial titers relative to Col‐0, whereas the overexpression lines showed increased bacterial titers under pretreated with flg22 treatment (Figure 4B). Next, MAPK phosphorylation levels were evaluated to assess the effect of AtIMPA1 on early apoplastic PTI. The specificity of the anti‐p44/42 ERK antibody was confirmed using the *fls2* mutant, which is defective in flg22‐induced MAPK activation. Consistent with this, the antibody detected strong phosphorylation signals in Col‐0 but no signals in the *fls2* mutant upon flg22 treatment. After flg22 treatment, *impa1* mutants exhibited significantly higher phosphorylation of MPK3/MPK6 than of Col‐0, whereas *AtIMPA1*‐overexpression lines showed reduced phosphorylation. The gray value analysis further supports the findings (Figure 4C). The callose deposition was further analyzed to assess the significance of AtIMPA1 in late PTI responses. The *impa1* mutants showed increased callose deposition after flg22 treatment, while the overexpression lines exhibited reduced callose deposition relative to Col‐0 (Figure 4D,E). Collectively, these results indicate that AtIMPA1 acts as a negative regulator of both basal and flg22‐triggered apoplastic defense.

**Figure 4:**
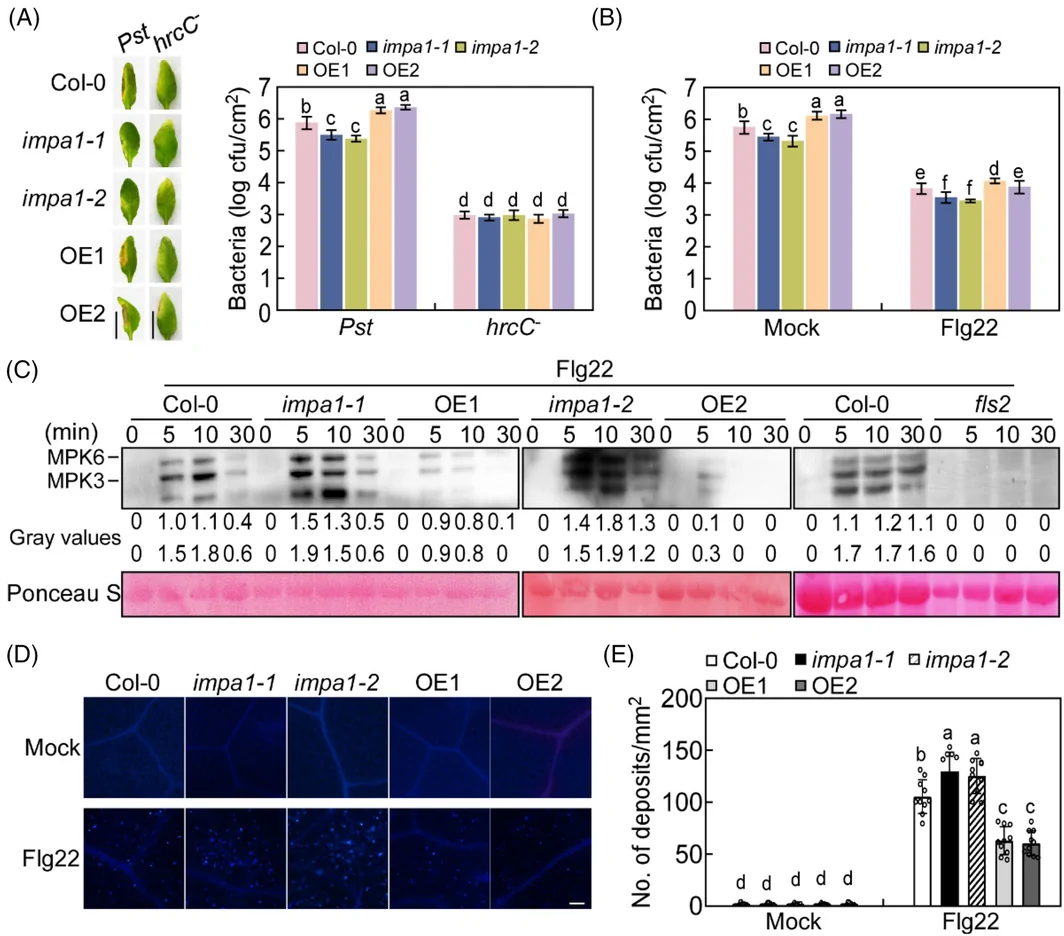
AtIMPA1 is involved in the apoplastic defense against *Pst* DC3000. (A) The disease symptoms and titers of 5‐week‐old Col‐0, *impa1‐1*, *impa1‐2* mutant, OE1, and OE2 plants were evaluated after syringe‐inoculated with *Pst* DC3000 (1 × 10^5^ cfu/mL) and *hrcC* ^−^(1 × 10^5^ cfu/mL), and photographed at 2 days post‐inoculation (dpi). Error bars of bacterial titers analysis are SDs of three biological replicates. Statistical analysis was performed by one‐way ANOVA with Tukey HSD using GraphPad Prism 8.0.1, and letters represents significant differences at *P* < 0.05. (B) The titers of 5‐week‐old Col‐0, *impa1‐1*, *impa1‐2* mutant, OE1, and OE2 plants were evaluated after syringe‐inoculated with *Pst* DC3000 (1 × 10^5^ cfu/mL) following pretreated with 100 nM flg22 or water (Mock) for 24  h. Error bars of bacterial titers analysis are SDs of three biological replicates. Statistical analysis was performed by one‐way ANOVA with Tukey HSD using GraphPad Prism 8.0.1, and letters represents significant differences at *P* < 0.05. (C) MAPK activation. 4‐week‐old Arabidopsis seedlings from Col‐0, *impa1* mutants and overexpression lines were infiltrated with 0.1 μM flg22. MAPK activation was analyzed by anti‐phosphor‐p44/42 MAK kinase immunoblotting. Experiments were repeated three times with similar results. The gray value analysis was analyzed by ImageJ. (D, E) The number of callose deposition were examined and statistically analyzed upon 100 nM flg22 in the 5‐week‐old Col‐0, *impa1‐1*, *impa1‐2* mutant, OE1, and OE2 plants. Leaf disks were stained with aniline blue and observed under a microscope after 24  h treated with flg22. Error bars are SDs of three biological replicates. Quantitative analysis of callose deposition was performed in 1 × 10^4^ μm^2^ from 10 fields in each lines using ImageJ. Statistical analysis was performed by one‐way ANOVA with Tukey HSD using GraphPad Prism 8.0.1, and letters represents significant differences at *P* < 0.05. Scale bar = 20 μm.

### AtIMPA1 modulated COR‐triggered stomatal reopening is linked to ABA signaling

To gain additional insight into global transcript changes caused by the mutation of AtIMPA1, whole‐transcriptome RNA‐Seq was conducted on Col‐0, the *impa1* mutant, and *AtIMPA1*‐OE. Differential expression analysis identified 985 differentially expressed genes (DEGs) in the *impa1‐1* mutant compared with Col‐0, with 478 genes upregulated and 507 genes downregulated. In *AtIMPA1*‐OE compared with Col‐0, 2280 DEGs were identified, comprising 877 upregulated and 1403 downregulated genes, and 2812 DEGs were found in the *impa1* mutant compared with *AtIMPA1*‐OE, with 1677 genes upregulated and 1135 genes downregulated (Figure S4A–C). Notably, cross‐comparison revealed 64 genes with opposite regulation patterns: 41 genes downregulated in *AtIMPA1*‐OE were upregulated in the *impa1* mutant, while 23 genes showed the inverse pattern (Figure S4E,F). GO enrichment analysis demonstrated that DEGs were significantly enriched in ABA‐responsive biological processes, particularly response to ABA and response to water deprivation (Figure S4D). Hierarchical clustering of well‐characterized ABA signaling genes demonstrated coordinated opposite regulation patterns between the *impa1* mutant and *AtIMPA1*‐OE (Figure S4G). These findings suggest that AtIMPA1 function is associated with ABA signaling, prompting us to investigate whether the involvement of AtIMPA1 in COR‐triggered stomatal reopening is mechanistically linked to ABA signaling. Firstly, the total ABA content in response to *Pst* DC3000 and *Pst* ^−^ were measured. Under mock conditions, basal ABA levels were indistinguishable among Col‐0, *impa1* mutants, and *AtIMPA1*‐overexpression lines. Upon inoculation with *Pst* DC3000 or the *Pst* ^−^ strain, total ABA content increased significantly in all genotypes. Notably, the *impa1* mutants accumulated less ABA than Col‐0, while the *AtIMPA1*‐overexpression lines exhibited higher ABA levels, particularly after *Pst* DC3000 inoculation (Figure 5A). Next, the stomatal aperture analysis showed that the stomata of Col‐0 were closed at 4 h upon ABA treatment, but they reopened following exposure to *Pst* DC3000 or ABA accompanied by *Pst* DC3000. In contrast, the stomata of overexpression lines remained closed when exposed to *Pst* DC3000, ABA or *Pst* DC3000 accompanied by ABA (Figure 5B). Fluridone is a well‐established inhibitor of ABA biosynthesis. Subsequently, fluridone was used to pretreat Col‐0 and *AtIMPA1*‐overexpression lines separately. Before bacterial treatment, leaves were pre‐incubated in stomatal opening buffer under light for 2 h to ensure a uniform open state, so that under mock conditions, stomata opened in all genotypes, regardless of whether they had been pretreated with mock or fluridone. Upon *Pst* DC3000 infection, COR‐induced stomatal reopening was observed in Col‐0 regardless of mock or fluridone pre‐treatment. In contrast, stomata of the *AtIMPA1*‐overexpression lines remained closed under mock pre‐treatment but opened following fluridone pre‐treatment. Crucially, when exogenous ABA was co‐applied with *Pst* DC3000 to the fluridone‐pretreated leaves, stomatal closure in the *AtIMPA1*‐overexpression lines was successfully restored. Furthermore, when challenged with the COR‐deficient mutant (*Pst* DC3000 COR^−^, *Pst* ^*−*^) or exogenous ABA alone, stomata of both genotypes remained closed under both mock and fluridone pre‐treatments (Figure 5C). Collectively, these results indicate that AtIMPA1 modulated COR‐triggered stomatal reopening is linked to ABA signaling.

**Figure 5:**
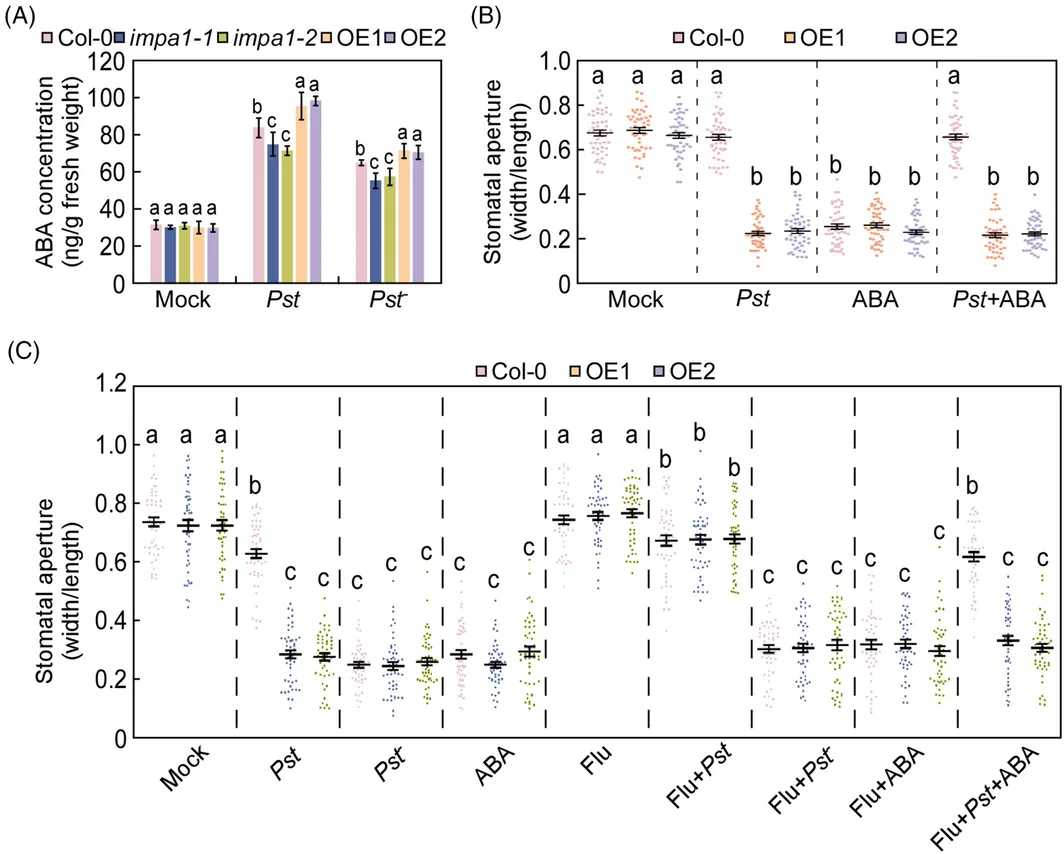
AtIMPA1 modulates coronatine (COR)‐triggered stomatal reopening is linked to abscisic acid (ABA) signaling. (A) ABA concentration was determined of 5‐week‐old Col‐0, *impa1‐1*, *impa1‐2* mutant, OE1, and OE2 plants were evaluated at 10 h after dip‐inoculated with *Pst* DC3000 (1 × 10^7^ cfu/mL) and *Pst* ^−^ (1 × 10^7^ cfu/mL). Statistical analysis was performed by one‐way ANOVA with Tukey HSD using GraphPad Prism 8.0.1, and letters represent significant differences at *P* < 0.01. (B) The stomatal aperture was statistically analyzed in Col‐0, OE1, and OE2 plants at 4  h after *Pst* DC3000 (*Pst*), 10 μM ABA or *Pst* DC3000 together with 10 μM ABA (*Pst* + ABA) treatment. Data are representative of three independent experiments with similar results. Quantitative analysis of stomatal aperture was performed from 50 stomata fields using ImageJ. Values represent average ± SEM. Statistical analysis was performed by one‐way ANOVA with Tukey HSD using GraphPad Prism 8.0.1, and letters represent significant differences at *P* < 0.01. (C) The stomatal aperture was statistically analyzed in Col‐0, OE1, and OE2 plants at 4  h after response to *Pst* DC3000, *Pst* ^−^ and ABA under fluridone pre‐treatment. ‘Flu’ represents the fluridone treatment. Data are representative of three independent experiments with similar results. Quantitative analysis of stomatal aperture was performed from 50 stomata fields using ImageJ. Values represent average ± SEM. Statistical analysis was performed by one‐way ANOVA with Tukey HSD using GraphPad Prism 8.0.1, and letters represent significant differences at *P* < 0.01.

### AtIMPA1 positively modulates ABA response

ABA could inhibit both seed germination and primary root elongation in Arabidopsis. We conducted seeding germination and root length assays to evaluate the effect of AtIMPA1 in response to ABA. The germination assays showed that all genotypes exhibited similar germination rates in the absence of ABA. Under 0.05 μM ABA treatment, the germination rates of the *impa1* mutants were significantly higher than those in the Col‐0, while the germination rates of the overexpression lines were markedly lower than those of Col‐0 (Figure 6A). The root length assays displayed that all genotypes exhibited similar growth rates under normal conditions and with 0.05 μM ABA treatment. However, when exposed to 0.07 μM ABA, the seedlings of the overexpression lines demonstrated a reduced growth rate, exhibiting root lengths approximately 9% shorter than those of the Col‐0. A more pronounced inhibition was noted at a concentration of 0.1 μM ABA, where the overexpression lines' seedlings displayed root lengths approximately 50% shorter than those of the Col‐0. In contrast, the seedlings of the *impa1* mutants exhibited longer root lengths than the Col‐0 seedlings under both 0.07 μM and 0.1 μM ABA treatments (Figure 6B). These results indicate that AtIMPA1 positively modulates ABA response in *Arabidopsis*.

**Figure 6:**
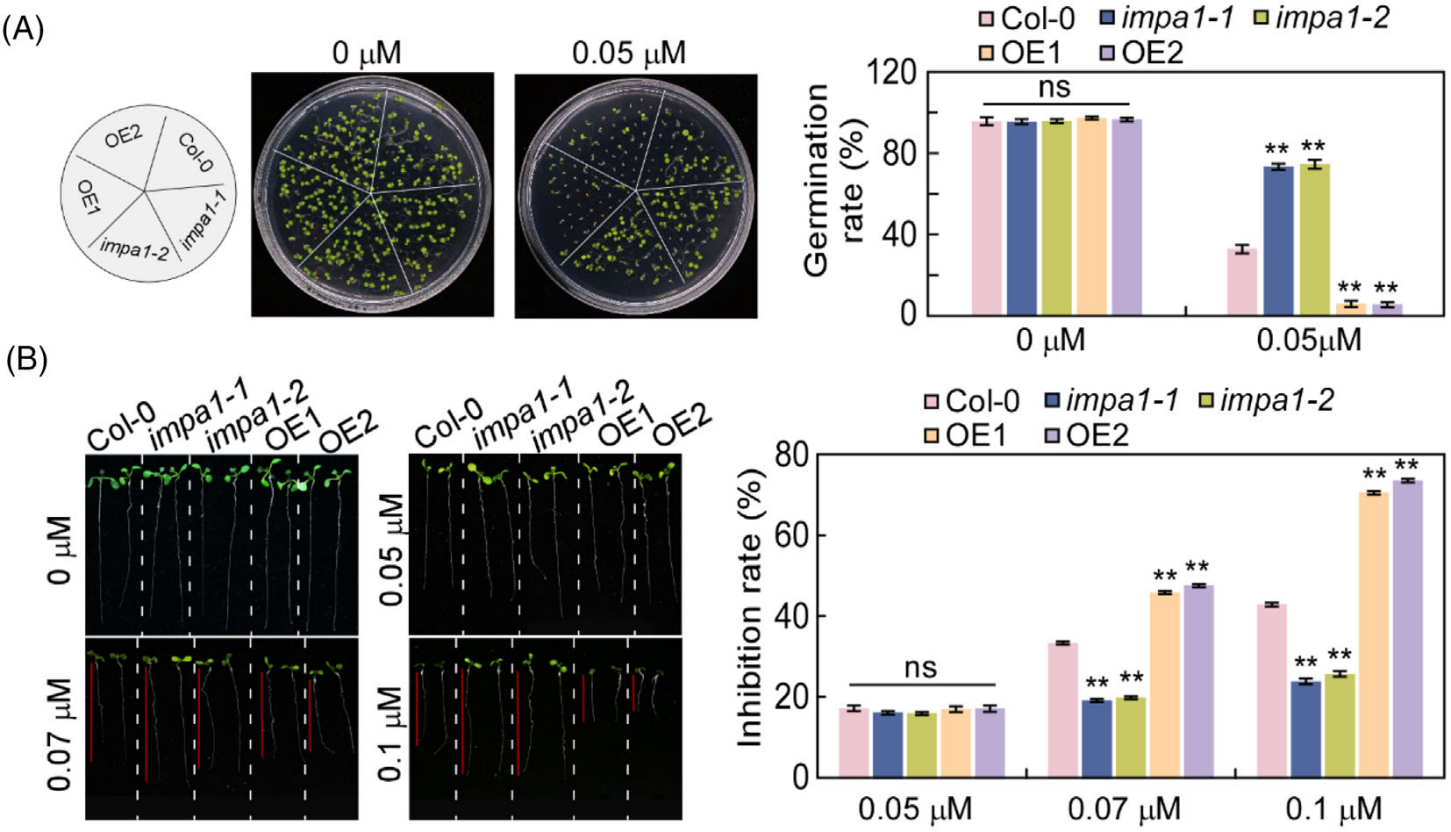
AtIMPA1 acts as a positive regulator in the abscisic acid (ABA)‐dependent pathway. (A) Seeds germination of Col‐0, *impa1‐1*, *impa1‐2*, OE1, and OE2 plants were evaluated and statistically analyzed on MS plates supplemented with 0 or 0.05 μM ABA for 72 h. Error bars are SDs of three biological replicates. Asterisks indicate significant differences by two‐tailed Student's *t*‐test using GraphPad Prism 8.0.1 at *P* < 0.01 (**). (B) Root lengths of all genotypes were evaluated and statistically analyzed on MS plates containing 0, 0.05, 0.07 and 0.1 μM ABA for 7 days. Error bars of bacterial titers analysis are SDs of three biological replicates. Asterisks indicate significant differences by two‐tailed Student's *t*‐test using GraphPad Prism 8.0.1 at *P* < 0.01 (**).

### Overexpression of the ABA‐responsive gene *AtARS1* in the *impa1* mutants can partially restore ABA insensitivity and disease‐susceptible phenotypes

To evaluate how AtIMPA1 influences downstream ABA signaling, AtARS1 (ABA and ROS SENSITIVE 1) was used as a molecular marker. AtARS1 is a well‐documented ABA‐responsive protein whose expression is significantly downregulated in the *impa1* mutant. Western blot analysis showed that while ABA treatment strongly induces AtARS1 protein accumulation in Col‐0, this accumulation is significantly compromised in the *impa1* mutant (Figure S5). Furthermore, the ABA‐triggered subcellular translocation of AtARS1‐GFP in mesophyll protoplasts was examined. The classic ABA‐induced cytoplasmic and membrane translocation of AtARS1 was significantly impaired in the *impa1* mutant (Figure S6B). As a critical control, AtABI5 (ABA Insensitive 5), another ABA‐responsive factor containing a cNLS, remained predominantly nuclear in both Col‐0 and *impa1* mutant, regardless of ABA treatment (Figure S6A). This altered localization of AtARS1 was further confirmed in stable transgenic plants (Figure S6C). Given that AtARS1 possesses a cNLS, the interaction between AtIMPA1 and AtARS1 was tested. However, yeast two‐hybrid (Y2H), bimolecular fluorescence complementation (BiFC), and co‐immunoprecipitation (Co‐IP) assays consistently failed to detect any direct interaction (Figure S7). Collectively, these results imply that the dramatically altered dynamics of AtARS1 in the *impa1* mutant are secondary consequences of a profound repression in the general upstream ABA signaling.

The *ars1* single mutant exhibited an immunocompromised phenotype similar to the *impa1* mutant, marked by severe bacterium‐mediated yellowing, elevated *Pst* DC3000 titers, and exacerbated COR‐triggered stomatal reopening (Figure 7A,B). To genetically assess whether AtARS1 functions within the AtIMPA1‐mediated pathway, *AtARS1‐GFP* was constitutively overexpressed in the *impa1* mutants, generating *AtARS1*‐OE/*impa1‐1* and *AtARS1*‐OE/*impa1‐2* lines. These lines exhibited intermediate disease resistance, with reduced symptom severity and bacterial titers compared with *impa1* single mutants, although not fully restored to Col‐0 level. Conversely, overexpression of *AtARS1* in Col‐0 (*AtARS1*‐OE/Col‐0) resulted in enhanced disease resistance and reduced bacterial titers compared with Col‐0 (Figure 7C). Consistent with these findings, seed germination assays showed that *AtARS1*‐OE/*impa1* seeds displayed intermediate ABA sensitivity, with germination rates higher than *impa1* but lower than Col‐0 (Figure 7D). These results demonstrate that constitutively expressing the downstream ABA‐responsive gene *AtARS1* can partially bypass the defect of AtIMPA1, providing evidence that AtIMPA1 functions upstream of AtARS1 in orchestrating ABA signaling for stomatal immunity.

**Figure 7:**
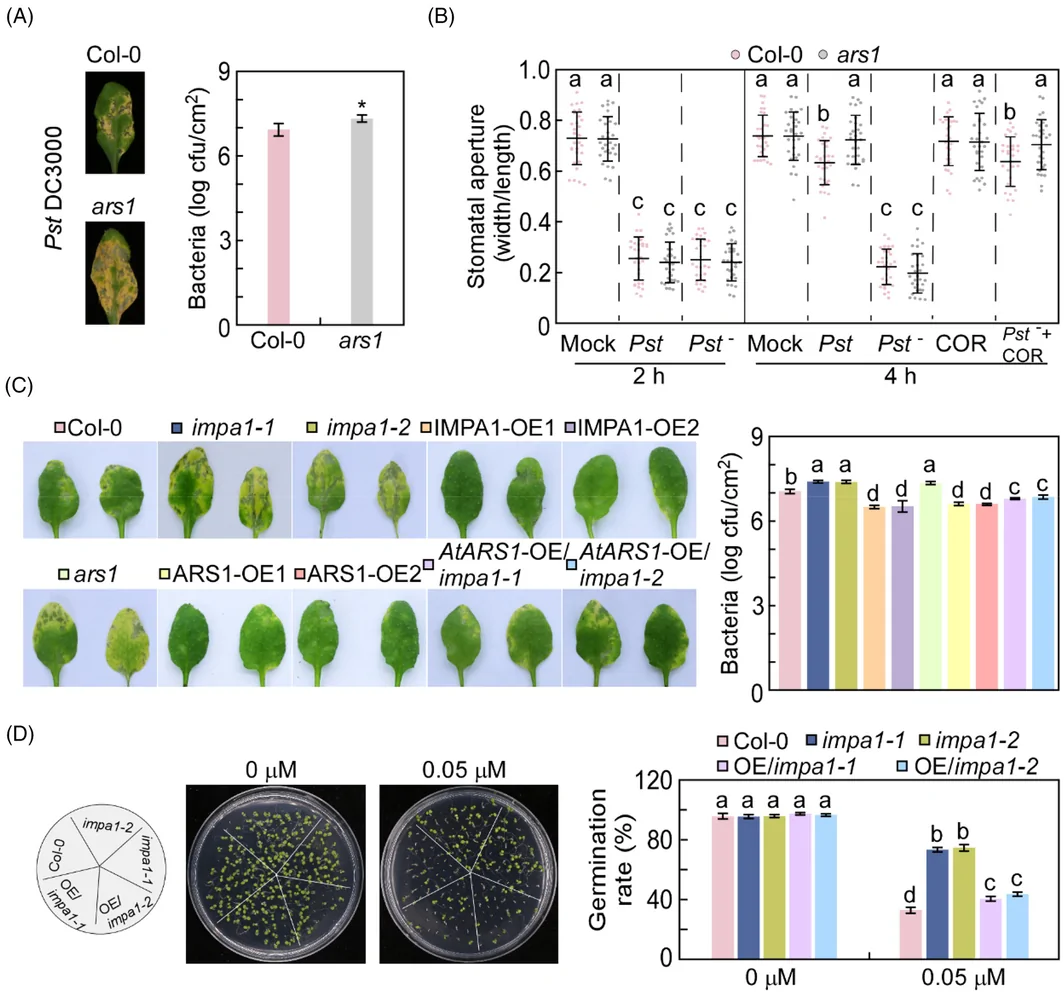
AtIMPA1 is required for AtARS1 in regulating abscisic acid (ABA) signaling and stomatal immunity. (A) The disease symptoms and titers of 5‐week‐old Col‐0 and *ars1* mutant were evaluated after dip‐inoculated with *Pst* DC3000 (1 × 10^7^ cfu/mL). Error bars of bacterial titers analysis are SDs of three biological replicates. Asterisks indicate significant differences by two‐tailed Student's *t*‐test using GraphPad Prism 8.0.1 at *P* < 0.05 (*). (B) The stomatal aperture were examined and statistically analyzed in Col‐0 and *ars1* mutant after *Pst* DC3000, *Pst* ^−^, coronatine (COR), and *Pst* ^−^ accompanied by COR treatment. Data are representative of three independent experiments with similar results. Quantitative analysis of stomatal aperture was performed from 30 stomata fields using ImageJ. Values represent average ± SEM. Statistical analysis was performed by one‐way ANOVA with Tukey HSD using GraphPad Prism 8.0.1, and letters represents significant differences at *P* < 0.05. (C) The disease symptoms and titers of 5‐week‐old Col‐0, *impa1* mutant, *AtIMPA1*‐overexpression lines, *ars1* mutant, *AtARS1*‐overexpression lines, and *AtARS1*‐OE/*impa1* plants were evaluated after dip‐inoculated with *Pst* DC3000 (1 × 10^7^ cfu/mL). Error bars are SDs of three biological replicates. Statistical analysis was performed by one‐way ANOVA with Tukey HSD using GraphPad Prism 8.0.1, and letters represents significant differences at *P* < 0.05. (D) Seeds germination of Col‐0, *impa1* mutant and *AtARS1*‐OE/*impa1* plants were evaluated and statistically analyzed on MS plates supplemented with 0 or 0.05 μM ABA. Error bars are SDs of three biological replicates. Statistical analysis was performed by one‐way ANOVA with Tukey HSD using GraphPad Prism 8.0.1, and letters represents significant differences at *P* < 0.01.

### AtIMPA1 contributes to ABA‐associated plant response to diverse pathogens

As a central phytohormone coordinating stress adaptation, ABA plays divergent functions contingent upon the lifestyle of the pathogen. The regulation of stomatal reopening by AtIMPA1, which contributes to defense against the bacterial pathogen *Pst* DC3000, implicates ABA signaling in modulating responses to distinct pathogen lifestyles. Necrotrophic fungus *Sclerotinia sclerotiorum* and the hemibiotrophic fungus *Colletotrichum higginsianum* were employed to investigate whether the ABA‐signaling deficiency observed in the *impa1* mutant manifests similarly across different pathogens. Interestingly, contrasting phenotypes were observed. Upon inoculation with *S. sclerotiorum*, the *impa1* mutants showed enhanced resistance compared with the Col‐0, whereas the *AtIMPA1*‐overexpression lines displayed compromised resistance with larger necrotic lesions. In sharp contrast, when challenged with *C. higginsianum*, the *impa1* mutants exhibited significantly decreased resistance, while the *AtIMPA1‐*overexpression lines showed enhanced resistance (Figure S8). Collectively, these results indicate that AtIMPA1 contributes to ABA‐associated plant response to different pathogens.

## DISCUSSION

In this study, we demonstrated that the nuclear importin α1 AtIMPA1 positively modulates the resistance to *Pst* DC3000 by modulating the stomatal reopening, primarily by compromising the COR‐triggered reopening of closed stomata induced by ABA. This study addressed the relationships among the nuclear importin α proteins, ABA signaling, and COR‐mediated stomatal immunity, and provides evidence that the transport mediated by nuclear importin α proteins is a finely tuned mechanism that contributes to resistance against pathogens in plants.

Importins in eukaryotic cells mediate nucleocytoplasmic trafficking by functioning as nuclear transport receptors that facilitate the transport of cytoplasmic proteins containing NLS (Xiong et al., 2021). Importins typically consist of a cargo‐binding α‐subunit (importin α), which recognizes cytosolic proteins containing cNLS, and a cargo‐navigating β‐subunit (importin β), which facilitates the transport of these cytosolic proteins into the nucleus (Xiong et al., 2021; Yang, He, et al., 2023). In this study, we identified the importin α protein AtIMPA1 and demonstrated that the mutation of *AtIMPA1* results in a significant reduction in the nuclear accumulation of a model cNLS‐RFP protein. Importin α proteins typically shuttle between the nucleus and cytoplasm. We performed time‐course observations of AtIMPA1‐GFP localization in *Arabidopsis* mesophyll protoplasts and *N. benthamiana* over a period ranging from 5 min to 2.5 h under ABA treatment or pathogen infection. During these observations, the GFP signal consistently remained nuclear. The predominantly nuclear signal of AtIMPA1‐GFP observed under steady‐state conditions has been documented for other plant importin α proteins, such as AtIMPA4 (Liu, Afrin, & Pajerowska‐Mukhtar, 2019; Liu, Xiong, et al., 2019) and wheat HvIMP α1 or HvIMP α2 (Wang et al., 2023). Numerous studies have documented the role of importin β proteins in plant immunity (Tamura & Hara‐Nishimura, 2014; Xu et al., 2013; Zheng et al., 2020); however, there is limited evidence regarding the involvement of importin α proteins in this process. Arabidopsis Importin α3/MOS6 is the widely studied protein that is required for basal plant immunity and the constitutive disease resistance activated in the auto‐immune mutant *snc1* (SUPPRESSOR OF NPR1‐1, CONSTITUTIVE 1) (Ludke et al., 2021; Palma et al., 2005). This study has thus increased the number of plant immunity players and suggests that the importin α protein is involved in the stomatal responses to bacteria.

Previous studies have shown that AtIMPA1 directly interacts with Arabidopsis importin β protein AtKPNB1. Inactivation of AtKPNB1 increased sensitivity to ABA during seed germination, increased stomatal closure in response to ABA, reduced the rate of water loss, and substantially enhanced drought tolerance (Luo et al., 2013). We documented that the *impa1* mutants exhibit decreased sensitivity to ABA during seed germination and root elongation, as well as impaired ABA‐induced stomatal closure. These findings provide substantial evidence that the AtIMPA1 positively modulates ABA response. While AtIMPA1 and AtKPNB1 typically function as an import complex, their opposing ABA phenotypes suggest that they may also have independent, potentially antagonistic roles in ABA signaling. AtARS1 encodes the ABA and ROS SENSITIVE 1 protein, which features two cNLS domains and is translocated to the cytoplasm and cell membrane in response to ABA (Baek et al., 2015). In this study, the mutation of *AtIMPA1* impaired protein accumulation and translocation of AtARS1 in response to ABA. Surprisingly, no direct physical interaction between AtIMPA1 and AtARS1 was detected. This indirect regulation parallels other well‐documented cases of plant nucleocytoplasmic transport. For instance, the Arabidopsis immune receptor SNC1 requires the nucleoporin MOS7 and the importin α3 homolog MOS6 for its nuclear accumulation and function, despite lacking direct physical interaction with these transport components (Cheng et al., 2009; Ludke et al., 2021; Palma et al., 2005). Similarly, while the importin β protein SAD2 typically mediates the nuclear import of MYB7, upon ABA treatment, the ARTA protein competitively hijacks SAD2, thereby blocking MYB7 from entering the nucleus and reducing its nuclear accumulation (Yang, He, et al., 2023). Given the role of AtIMPA1 in nuclear importin, it might be that AtIMPA1 was responsible for the nuclear import of an upstream signaling component, such as ABA‐responsive kinase or phosphatase, which modifies AtARS1 to alter its relocation. Alternatively, an intermediate adaptor protein might bridge AtIMPA1 and AtARS1 in a dynamic complex. Importantly, introducing the *AtARS1‐OE* construct into the *impa1* mutant partially restored ABA insensitivity and disease‐susceptible phenotypes to *Pst* DC3000, indicating that AtARS1 acts downstream of AtIMPA1. The incomplete complementation suggests either that other AtIMPA1 cargo proteins contribute to the full phenotype, or that the AtARS1 protein accumulation achieved by constitutive overexpression cannot fully compensate for the dynamic, stimulus‐responsive import normally mediated by AtIMPA1.

Plant resistance to *Pst* DC3000 comprises two spatially and temporally distinct phases: pre‐invasion stomatal immunity and post‐invasion apoplastic defense (Melotto et al., 2006). In dipping inoculation, which engages both defense layers, *impa1* mutants were highly susceptible to *Pst* DC3000, while overexpression lines showed enhanced resistance. However, in syringe‐infiltration, which bypasses stomata to assess apoplastic defense independently, *impa1* mutants exhibited increased resistance. Given these opposing effects, the overall resistance phenotype observed in dip‐inoculation strongly indicates that the predominant contribution of AtIMPA1‐mediated stomatal immunity overrides its antagonistic effect on apoplastic defense. Differences between dip‐ and syringe‐ inoculation were also observed in other mutants/overexpression lines defective in bacteria‐induced stomatal immunity. Overexpression of the ABA receptor, RCAR3, enhanced susceptibility to *Pst* DC3000 after syringe‐infiltration, while RCAR3‐overexpression lines displayed enhanced resistance against bacterial infection in dipping inoculation by inhibiting stomatal reopening (Lim et al., 2014). In terms of the *impa1* mutants, an increased resistance to syringe‐inoculation *Pst* DC3000 was observed; however, resistance to the *Pst* DC3000 *hrcC* ^−^ strain did not exhibit a similar enhancement. This is consistent with previous studies showing that plant importin α proteins can facilitate nuclear trafficking of bacterial effectors. Peng *et al*. and Hui *et al*. have documented that rice importin α1 (IMPα1b) negatively modulates resistance to *Xoo* by mediating the nuclear trafficking of bacterial type III effectors within plant cells (Hui et al., 2019; Peng et al., 2022). Similarly, we propose that AtIMPA1 facilitates nuclear trafficking of one or more *Pst* DC3000 T3 effectors that suppress apoplastic immunity. The observation that the *impa1* mutant is susceptible to *Pst* DC3000 and *C. higginsianum* but resistant to the aggressive necrotroph *S. sclerotiorum* clearly demonstrates that AtIMPA1 does not confer broad‐spectrum, non‐specific basal resistance. Instead, it specifically modulates early defense responses, such as stomatal immunity, which are crucial for restricting hemibiotrophic and early‐stage biotrophic infection. Phenotypic differences were observed between the *AtIMPA1* complementation and overexpression lines. These phenotypic variations can be attributed to differences in the expression levels of AtIMPA1. Specifically, the complementation lines merely restored *AtIMPA1* expression to Col‐0 levels, whereas the overexpression lines accumulated substantially higher AtIMPA1 transcript and protein. While basal AtIMPA1 levels in Col‐0 and complemented plants confer basic defense, elevated AtIMPA1 abundance in the overexpression lines significantly enhances the capacity to sustain stomatal closure during *Pst* DC3000 infection.

COR is a virulence factor in the infection of Arabidopsis by *Pst* DC3000, and it can reopen closed stoma, thereby promoting the entry of the pathogen (He et al., 2012; Lim et al., 2014). It has been reported that COR and the ABA‐responsive protein are synergistically involved in plant immunity. The ABA‐signaling component, HAI1, a clade‐A PP2C protein, dephosphorylated MPK3 and MPK6, which is necessary for COR‐mediated suppression of MPK3/MPK6 activation and immunity (Mine et al., 2017). In this study, we investigated the regulation of stomatal dynamics by AtIMPA1 under direct exposure to *Pst* DC3000, ABA and *Pst* DC3000 accompanied by ABA treatment, as well as the effects of exogenous application of fluridone, and found that AtIMPA1 regulates COR‐mediated stomatal reopening by orchestrating ABA signaling, similar to the effect observed with the ABA receptor RCAR3 (Lim et al., 2014). Recent data have demonstrated that importin β protein (SAD2) imports ABI1 (a component of ABA signaling) to the plant nucleus. COR orchestrates SAD2 and ABI1 to open plant stomata during pathogen invasion (Liu et al., 2024). These findings provide compelling evidence of a significant relationship among nuclear importin α proteins, ABA signaling, and COR‐mediated stomatal immunity.

In conclusion, we propose a working model outlining how the nuclear importin α1 AtIMPA1 modulates stomatal reopening to defend against *Pst* DC3000 by orchestrating ABA signaling (Figure 8). *Pst* DC3000 infection triggers ABA accumulation, which induces rapid stomatal closure to restrict pathogen entry. However, the pathogen *Pst* DC3000 deploys the toxin COR to counteract ABA‐induced stomatal closure, promoting the reopening of closed stomata. In this context, AtIMPA1 compromises the COR‐triggered reopening of closed stomata induced by ABA, thereby reinstating this closure. AtIMPA1 modulates expression levels of ABA‐related proteins, such as AtARS1, potentially enhancing the plant's adaptability to pathogens. Based on the results above, it's interesting to explore which cargo proteins collaborate with AtIMPA1 in the process of COR‐triggered stomatal reopening, as this would provide deeper mechanistic insight into AtIMPA1's role in plant immunity.

**Figure 8:**
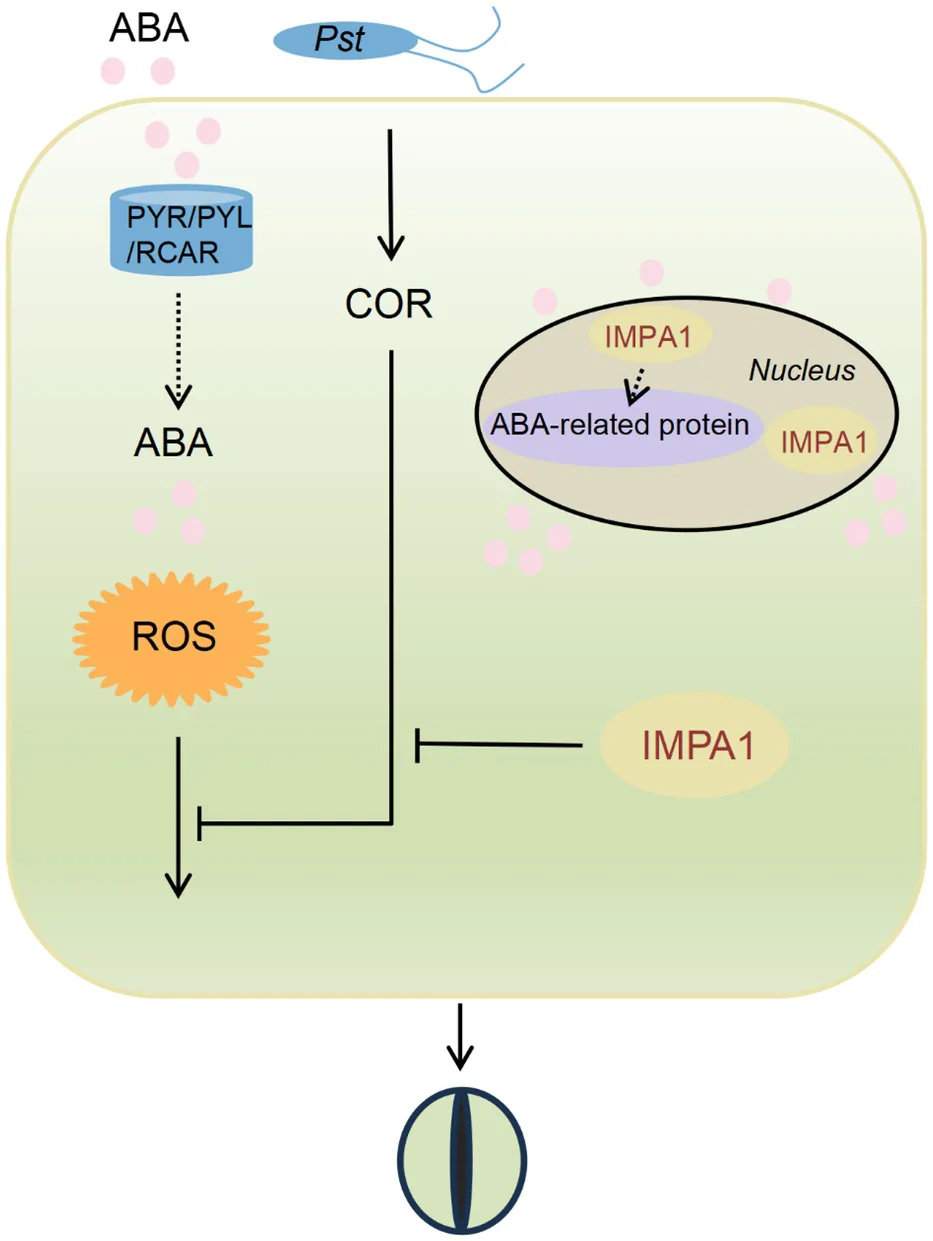
Proposed model in which nuclear importin α1 AtIMPA1 modulates stomatal reopening to defend against *Pst* DC3000 by orchestrating abscisic acid ABA signaling. *Pst* DC3000 infection triggers ABA accumulation, which induces rapid stomatal closure to restrict pathogen entry. However, the pathogen *Pst* DC3000 deploys the toxin COR to counteract ABA‐induced stomatal closure, promoting the reopening of closed stomata. In this context, AtIMPA1 compromises the COR‐triggered reopening of closed stomata induced by ABA, thereby reinstating this closure. Additionally, functioning as a nuclear importin α1 protein, AtIMPA1 modulates expression levels of ABA‐related proteins, such as AtARS1, potentially enhancing the plant's adaptability to pathogens.

## MATERIALS AND METHODS

### Plant material and growth conditions

*A. thaliana* Columbia (Col‐0) was used as the wild‐type in this study. The *impa1‐1* mutant (SALK_105557) and *impa1‐2* mutant (SALK_082616) were obtained from Arabidopsis Biological Resource Center (https://abrc.osu.edu). To generate *AtIMPA1* overexpression lines, the full‐length AtIMPA1 coding sequence was cloned into the pCN‐GFP vector under control of the CaMV 35S promoter. For complementation lines, the same coding sequence was cloned into pCN‐GFP driven by the native *AtIMPA1* promoter (a 945‐bp genomic sequence upstream of the ATG start codon). The resulting constructs were transformed into Arabidopsis Col‐0 (for overexpression) or *impa1* mutants (for complementation) using the *Agrobacterium tumefaciens* GV3101‐mediated floral dip method (Clough & Bent, 1998). Seedlings of *A. thaliana* were grown on solidified half‐strength Murashige and Skoog (MS) medium supplemented with 1% sucrose for 2 weeks. *Arabidopsis* plants were grown in commercial potting soil with perlite in a 3:2 ratio, at 22°C, under 80 mE m^−2^ s^−1^ illumination using bulb‐type fluorescent light with 10 h‐ light/14 h‐night photoperiod.

### Pathogen infection assays

*Pst* DC3000, *Pst* ^−^ and *Pst* DC3000 *hrcC* ^−^ strains were cultured in King's B medium with kanamycin at 28°C overnight, collected, and resuspended in 10 mM MgCl_2_. 5‐week‐old plants were dip‐inoculated with *Pst* at a concentration of 1 × 10^7^ cfu/mL in 0.02% (v/v) Silwet‐77 or syringe‐inoculated at a concentration at 1 × 10^5^ cfu/mL, respectively. Diseased leaves were photographed at 2 or 3 dpi. Leaf samples collected after infection, homogenized, and subjected to gradient dilutions as described previously (Song et al., 2022). For fungal pathogen infection assays, mycelial plugs of *S. sclerotiorum* or *C. higginsianum* were placed on fully expanded leaves of 5‐week‐old Arabidopsis plants. Disease symptoms and lesion diameters were determined at 24 hpi for *S. sclerotiorum* and at 3 dpi for *C. higginsianum*.

### Reverse‐transcription quantitative real‐time PCR

Reverse transcriptase‐quantitative polymerase chain reaction (RT‐qPCR) assay was performed as described in a previous study (Vogels et al., 2020). Leaves from 5‐week‐old plants were spray inoculated with mock or *Pst* DC3000 and were harvested at indicated times after inoculation. RNA samples were extracted from infected leaves using the RNAsimple Total RNA Kit (TIANGEN, Beijing, China). cDNA was prepared using reverse transcriptase HiScript III RT SuperMix for qPCR (Vazyme Biotech, Nanjing, China). RT‐qPCR was run on an Applied Biosystems 7500 real‐time PCR system (Foster City, CA, USA) with SYBR Premix ExTaq (Vazyme Biotech, Nanjing, China). The relative quantification of each transcript was calculated by the 2^‐ΔΔCt^ method. The RT‐qPCR assay was repeated three times with three biological replicates. The primers used in this section are listed in Table S1.

### Subcellular localization

To observe the localization of AtIMPA1, the full‐length coding sequences of the *AtIMPA1* gene were cloned into the pCN‐GFP vector and its expression was driven by the *AtIMPA1* native promoter, which was defined and amplified as the 945‐bp genomic DNA fragment immediately upstream of the start codon (ATG). This recombinant construct was then transformed into *Arabidopsis* Col‐0 plants using the *Agrobacterium tumefaciens* GV3101‐mediated floral dipping method (Clough & Bent, 1998). The cNLS‐RFP construct served as a nuclear localization marker in the subcellular localization assay. This construct employs the classical SV40 NLS, which has the amino acid sequence PKKKRKV, encoded by the nucleotide sequence CCTAAGAAGAAGAGAAAGGTT (Adam et al., 1989). A DNA fragment containing three tandem repeats of this sequence was cloned into the pUC19‐RFP vector driven by the CaMV 35S promoter. The pUC19‐AtIMPA1‐GFP, pUC19‐AtABI5‐GFP, pUC19‐AtARS1‐GFP, and pUC19‐cNLS‐RFP were transformed into *Arabidopsis* mesophyll protoplasts, as described previously (Luo et al., 2013). Fluorescence signals of GFP were quantified with ImageJ as described previously (Zhang et al., 2022). The pBINPLUS‐AtIMPA1‐GFP was introduced into *A. tumefaciens* strain GV3101 and infiltrated into fully expanded leaves of 5‐week‐old *Nicotiana benthamiana*. DAPI staining was used to define nuclear boundaries. The resulting transformants were observed under a confocal fluorescence microscope (AX, 480 20x; Nikon).

### Stomatal aperture assay

Leaves from 5‐week‐old *Arabidopsis* plants were harvested and flooded on approximately 20 mL of freshly prepared stomatal buffer (50 mM KCl, 50 μM CaCl_2_ and 10 mM MES‐Tris, pH 6.15) and kept under light for at least 2 h to open stomata before the beginning of the experiments. Specifically, for the fluridone pre‐treatment assay, leaves were incubated in the stomatal buffer supplemented with either 10 μM fluridone or a mock solution during this 2 h light period to ensure uniform stomatal opening. Subsequently, the leaves were treated with 1 μM flg22, 10 μM ABA, bacteria (1 × 10^8^ cfu/mL), 2 μM COR, or co‐applied with combinations thereof, as described previously (Song et al., 2022; Wang, Song, et al., 2024). The peels were mounted on slides and observed under a microscope (ECLIPSE Ti2, 40x; Nikon). For each sample, epidermal peels were collected from at least three individual plants, and a minimum of 50 stomata were examined. Stomatal aperture was equal to the width/length ratio of the stomata opening.

### MAP kinase assay

MAP kinase assay was performed as described previously (Nitta et al., 2020). The 4‐week‐old Arabidopsis leaves of Col‐0, the *impa1* mutants, and overexpression lines were collected at 0, 10, and 30 min after infiltrated with 0.1 μM flg22. The samples were lysed in lysis buffer (50 mM Tris–HCl, pH 7.5, 150 mM NaCl, 0.5 mM EDTA, 0.5% Nonidet P‐40, and 1× protease inhibitor cocktail). Crude extracts were centrifuged at 12000 rpm for 20 min at 4°C, and the supernatants were collected. Proteins were separated by 10% SDS‐PAGE gel and then transferred onto polyvinylidene fluoride membrane. Phosphorylated MAP kinases were detected by Western blot using the anti‐p44/42‐ERK antibody (Cell Signaling Technology). The *fls2* mutant served as a negative control to test the specificity of the anti‐p44/42‐ERK antibody.

### Callose deposition assay

For callose deposition assay, the leaves from 5‐week‐old Arabidopsis were infiltrated with 100 nM flg22 and by double‐distilled water only for the mock controls. Cleared leaf disks were stained with 0.01% (w/v) aniline blue in 0.15 M phosphate buffer, pH 9.5, for 4 h. Callose deposits were visualized under UV light illumination using a microscope (ECLIPSE Ti2, 40x; Nikon). Quantification of callose deposits was performed on the acquired digital images using ImageJ.

### Seed germination and root lengths assays

For seed germination assays, seeds (at least 50 seeds for each genotype) harvested at approximately the same time were surface sterilized and sown on MS medium or MS medium containing 0.05 μM ABA in light at 22°C. The seedlings were photographed at 72 h. Germination rate was calculated as the proportion of germinated seeds from the total in each experiment. For the root lengths assays, all seedlings were grown on MS medium containing 0, 0.05, 0.07, and 0.1 μM ABA for 7 days in light at 22°C.

### Yeast two‐hybrid assay

The coding sequences of *AtIMPA1* and *AtARS1* were cloned into pGADT7 (AD) and pGBKT7 (BD) vectors, respectively. The constructs were co‐transformed into yeast strain AH109 and selected on SD/‐Leu/‐Trp medium. Positive transformants were subsequently spotted onto selective SD medium lacking leucine, tryptophan, histidine, and adenine (SD/‐Leu/‐Trp/‐His/‐Ade) and incubated at 30°C for 3 d.

### Co‐immunoprecipitation assay

AtARS1‐GFP and AtIMPA1‐Flag constructs were transiently co‐expressed in *N. benthamiana* leaves, with *35S‐GFP* as a negative control. After 48 h, total proteins were extracted and incubated with anti‐GFP beads at 4°C. The input and immunoprecipitated proteins were then separated by SDS‐PAGE and analyzed by immunoblotting using anti‐GFP and anti‐Flag antibodies.

### Bimolecular fluorescence complementation assay

*AtIMPA1* and *AtARS1* were fused to the N‐ or C‐terminal fragments of YFP. The resulting constructs were transformed into *Agrobacterium* strain GV3101, mixed, and co‐infiltrated into *N. benthamiana* leaves. After 48 h of incubation, the reconstituted YFP fluorescence was observed using a confocal microscope.

### ABA quantification

Five‐week‐old plants were dip‐inoculated with *Pst* DC3000 and *Pst* ^*−*^ at a concentration of 1 × 10^7^ cfu/mL in 0.02% (v/v) Silwet‐77. ABA levels were measured in all genotypes at 10 hpi (Liu, Afrin, & Pajerowska‐Mukhtar, 2019). ABA extraction and quantification were performed as previously described (Roussin‐Léveillée et al., 2022).

### Statistical analysis

Statistical analysis was performed with GraphPad Prism 8.0.1. Two‐tailed Student's *t*‐test and one‐way ANOVA were used to analyze data. The significance of differences between samples was statistically evaluated using SD or SEM.

## Conflict of Interest

The authors declare no potential conflict of interest.

## Supporting information


**Figure S1.** Identification of the *impa1* mutants, overexpression lines, and complemented lines.
**Figure S2.** Sequence alignment and phylogenesis analysis of AtIMPA1 protein.
**Figure S3.** AtIMPA1‐complemented lines fully rescue the *impa1* mutant phenotypes related to disease resistance and stomatal responses.
**Figure S4.** Transcriptome profiling of Col‐0, *impa1* mutant, and *AtIMPA1‐*overexpression lines.
**Figure S5.** AtARS1 protein levels in Col‐0 and the *impa1‐1* mutant following ABA treatment.
**Figure S6.** AtIMPA1 is involved in nuclear trafficking of AtARS1 under ABA treatment.
**Figure S7.** AtIMPA1 does not interact with AtARS1.
**Figure S8.** AtIMPA1 modulates resistance to *S. sclerotiorum* and *C. higginsianum*.
**Table S1.** Primers used in this study.

## Data Availability

All relevant data can be found within the manuscript and its supporting materials.
