# Supplementary material for: Nuclear importin α1 AtIMPA1 regulates stomatal reopening to defend against Pst DC3000 by orchestrating ABA signaling
Source: Plant J. 2026 Oct 29;128(3):e71175. doi: 10.1111/tpj.71175 (PMC13720327; doi:10.1111/tpj.71175)
Supplement: Supplementary file 1 [file TPJ-128-0-s001.pdf]

Nuclear importin α1 AtIMPA1 regulates stomatal reopening to defense against *Pst* DC3000 by orchestrating ABA signaling

Ting Zhang[1,2], Kang Wang[1,2], Yi Guan[1,2], Jingao Zhang[1,2], Charles Tetteh[1,2], Chenxi Liu[1,2], Cheng Zhang[1,2], Pinyuan Jin[1,2], Sheng Luo[1,2], Yiling Wang[1,2] and Huajian Zhang[1,2]*

Supplementary Materials

Table S1. Primers used in this study

Primer name	Sequence (5'-3')	Remark
IMPA1-LP1	CTTCTTCTCCAGTGCGTGATC	validation of *impa1-1* mutant line
IMPA1-RP1	AATAGCCCTGGACAATGAACC	
IMPA1-LP2	GTGCTTTCATTCTTTGGATGC	validation of *impa1-2* mutant line
IMPA1-RP2	TGTTTGTTAGAGCCCAAGCTG	
PBIN-IMPA1-F	GGTACCCCCGGGGTCGACATGTCACTGAGACCCAACG	AtIMPA1 localization
PBIN-IMPA1-R	TCTAGAGGTACCGTCGACGCTGAAGTTGAATCCTCCGG	
PUC19-IMPA1-F	AGAAGGCCTACTAGTGGATCCATGTCACTGAGACCCAACG	AtIMPA1 localization
PUC19-IMPA1-R	GGGGGTACCGTCGACGGATCCGCTGAAGTTGAATCCTCCGG	
PCNG-IMPA1-F	TCCGTCGACGGTACCCCCGGGATGTCACTGAGACCCAACG	AtIMPA1-overexpression
PCNG-IMPA1-R	ACCAGACCCGCCTCCCCCGGGGCTGAAGTTGAATCCTCCGG	
proIMPA1-F	TCCGTCGACGGTACCCCCGGGCATGGCGGGGAAAGTTC	*AtIMPA1*-GFP driven by its native promoter, used for analyzing the expression pattern of AtIMPA1
proIMPA1-R	ACCAGACCCGCCTCCCCCGGGGCTGAAGTTGAATCCTCCGG	
cNLS-RFP-F	ACGAATCTCAAGCAATCAAATGGCCTCCTCCGAGGACGTCAT	cNLS-RFP construct, used as a nuclear marker for subcellular localization assays
cNLS-RFP-R	GCCGTCCAGCTCGACCAGGAACCTTTCTCTTCTTCTTAG	
QPR1-F	AGGTGCTCTTGTTCTTCCCT	RT-qPCR analysis of *PR1*
QPR1-R	ACCCCAGGCTAAGTTTTCCC	
QNPR1-F	AAGTTTCTTTCCACCGGTGC	RT-qPCR analysis of *NPR1*
QNPR1-R	GGTCTCACTCTGCTGCTGTA	
QActin-F	CGCTGACCGTATGAGCAAAGAAAT	RT-qPCR analysis of *Actin*
QActin-R	CTGAGGGAAGCAAGAATGGAACC	

ABI5-GFP-F	AGAAGGCCTACTAGTGGATCCATGGTAACTAGAGAAACGAAG TTGACG	ABI5 localization
ABI5-GFP-R	GGGGGTACCGTCGACGGATCCGAGTGGACAACTCGGGTTCCT	
ARS1-LP1	AATGGTGAATGGAAACCTTCC	validation of *ars1* mutant line
ARS1-RP1	TTATCACCTGAAACTGGTCGG	
ARS1-GFP-F	AGAAGGCCTACTAGTGGATCCATGGATGCTCAAGCGAAGAAG AAG	ARS1 localization
ARS1-GFP-R	GGGGGTACCGTCGACGGATCCAAGATGTTGAGCTCTCCAATC AAC	

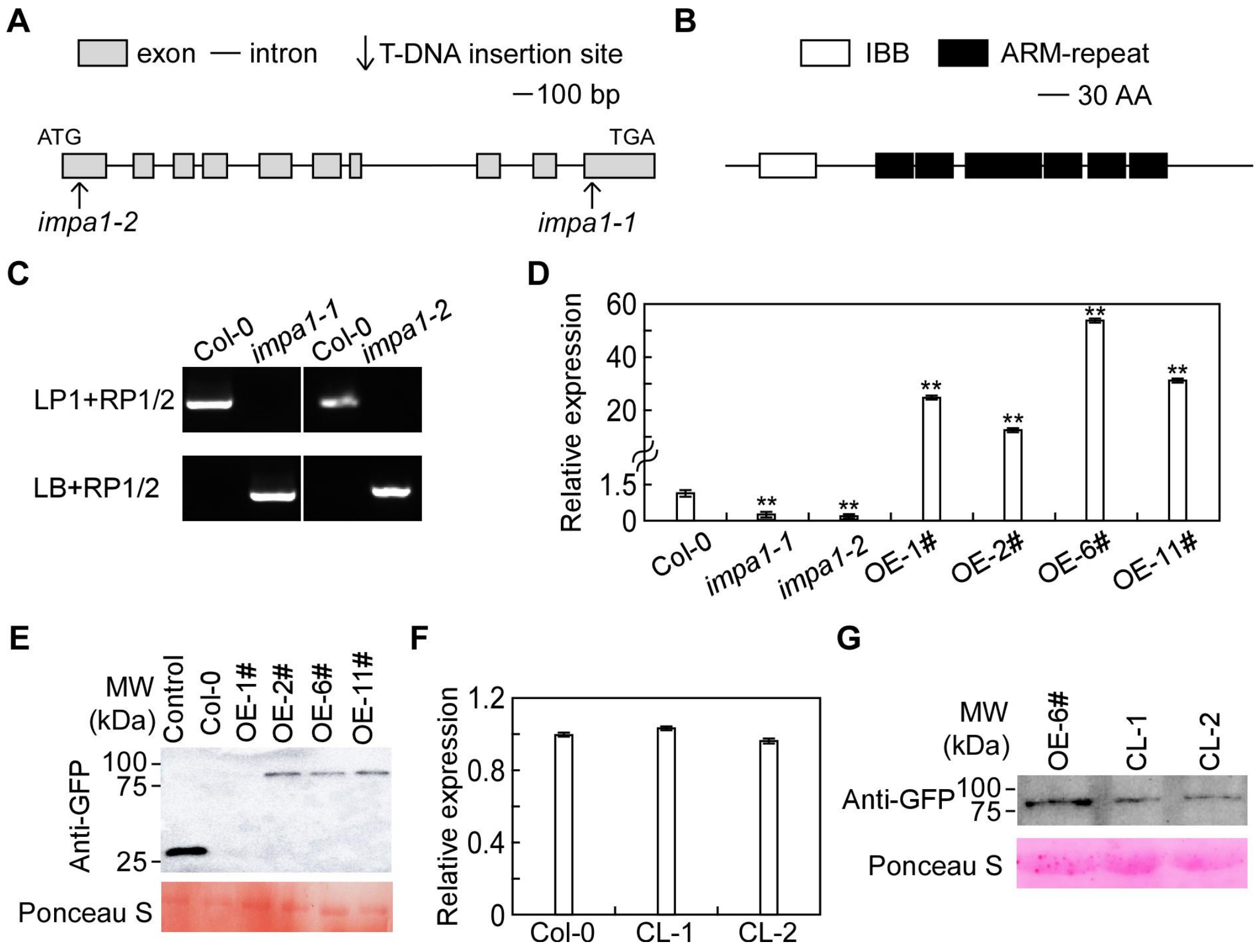

Figure S1. Identification of the *impa1* mutants, overexpression lines, and complemented lines. (A) Schematic representation of the *AtIMPA1* gene T-DNA insertion site. (B) Annotated AtIMPA1 protein structure. (C) The *impa1-1* and *impa1-2* T-DNA insertion mutants were verified by PCR. (D) *AtIMPA1* expression levels in Col-0, mutants and overexpression lines. (E) The overexpression lines were verified by western blot. The control is represented by empty pCN-GFP transformed into Col-0. (F) RT-qPCR analyses of the expression levels of the *AtIMPA1* gene in Col-0 and AtIMPA1-complemented lines (CL-1 and CL-2). (G) Western blot analyses of the protein levels of the AtIMPA1 in *AtIMPA1*-overexpression lines and *AtIMPA1*-complemented lines (CL-1 and CL-2).

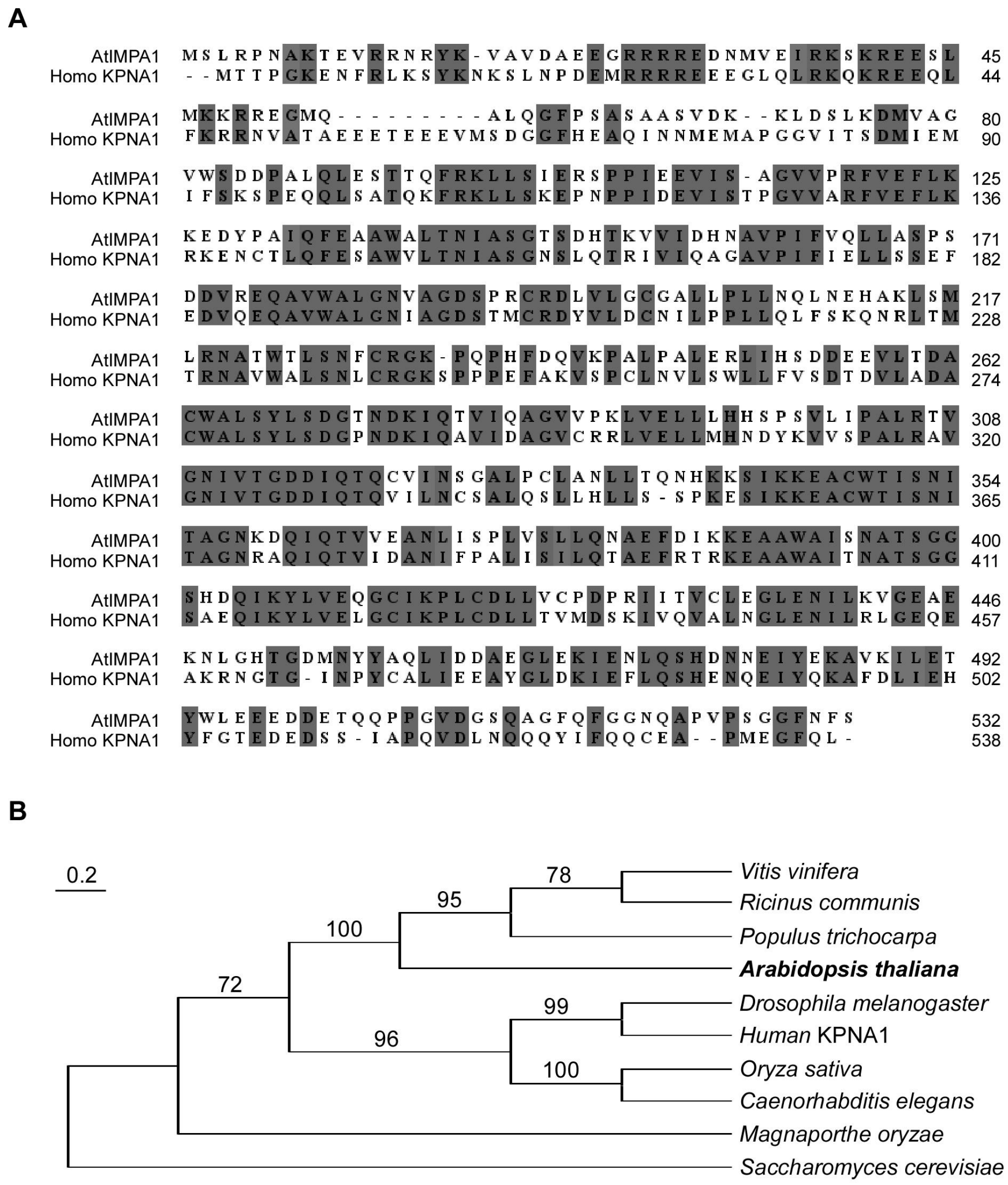

Figure S2. Sequence alignment and phylogenesis analysis of AtIMPA1 protein. (A) Sequence alignment of *A. thaliana* AtIMPA1 and human KPNA1 protein. (B) Phylogenetic trees of importin-α1 related proteins from several organisms were constructed using the CLUSTAL_W and MEGA 11 programs by the neighbor-joining method with 1000 bootstrap replicates. Species names and GenBank accession numbers are as follows: XP 010649542.1 (*Vitis vinifera*), XP 015574730.1 (*Ricinus communis*), XP 002318966.1 (*Populus trichocarpa*), NP 187328.1 (AtIMPA, *A. thaliana*), NP 524167.1 (*Drosophila melanogaster*), EAW79483.1 (*Human*), BAA31222.1 (*Oryza sativa*), AAB97173.1 (*Caenorhabditis elegans*), EHA55434.1 (*Pyricularia oryzae*) and pdb|1UN0|A (*Saccharomyces cerevisiae*).

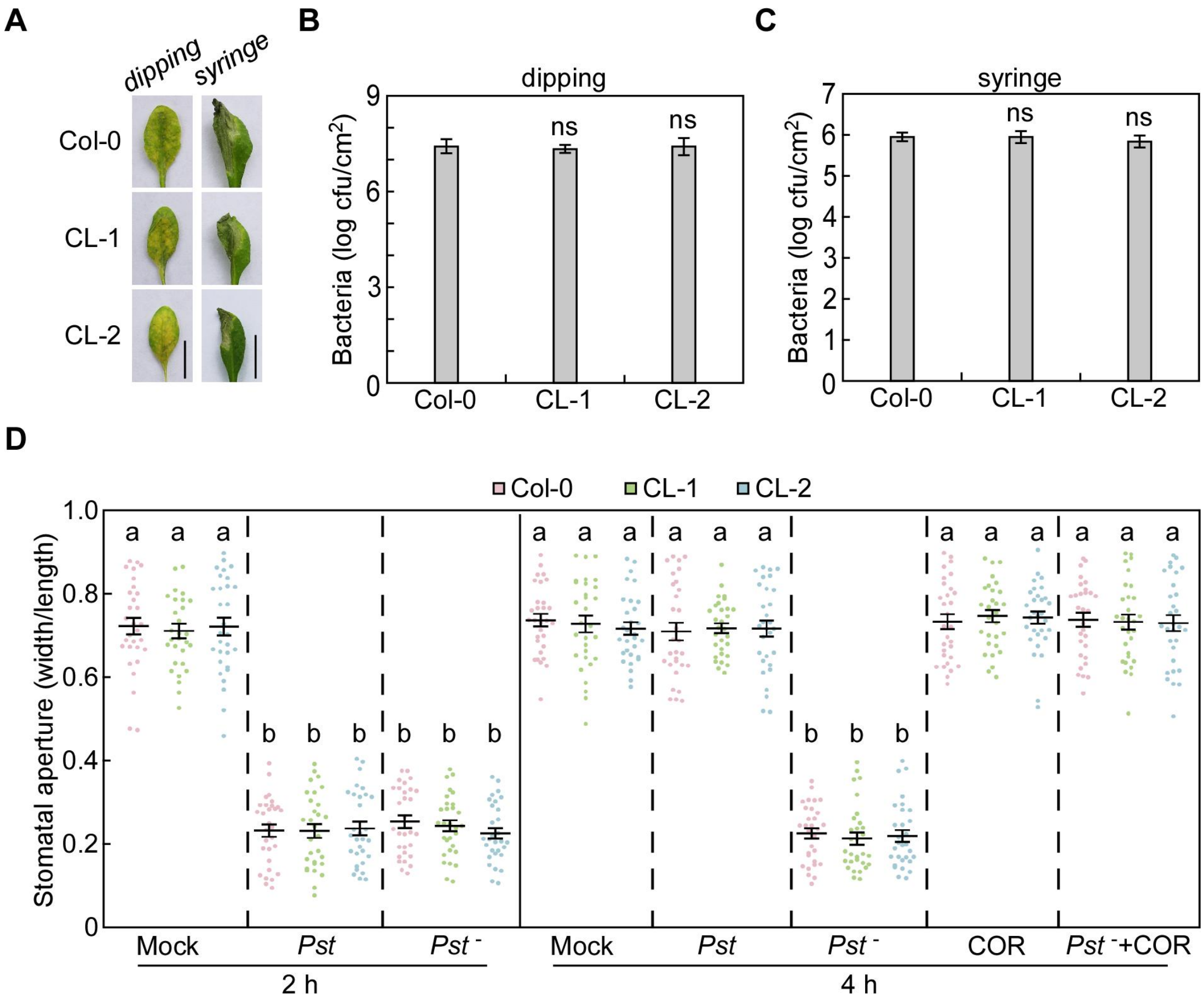

Figure S3. *AtIMPA1*-complemented lines fully rescue the *impa1* mutant phenotypes related to disease resistance and stomatal responses. (A, B and C) The disease symptoms and titers of 5-week-old Col-0, CL-1 and CL-2 plants were evaluated after dip- or syringe- inoculation with *Pst* DC3000. Error bars of bacterial titers analysis are SDs of three biological replicates. Scale bar = 1 cm. (D) The aperture of stomatal was examined and statistically analyzed in Col-0, CL-1 and CL-2 plants at 2 and 4 hours after *Pst* DC3000, *Pst*$^-$, COR, and *Pst*$^-$ accompanied by COR treatment. Quantitative analysis of stomatal aperture was performed from 30 stomata fields using ImageJ. Values represent average ± SEM. Statistical analysis was performed by one-way ANOVA with Tukey HSD using GraphPad Prism 8.0.1, and letters represents significant differences at P<0.01.

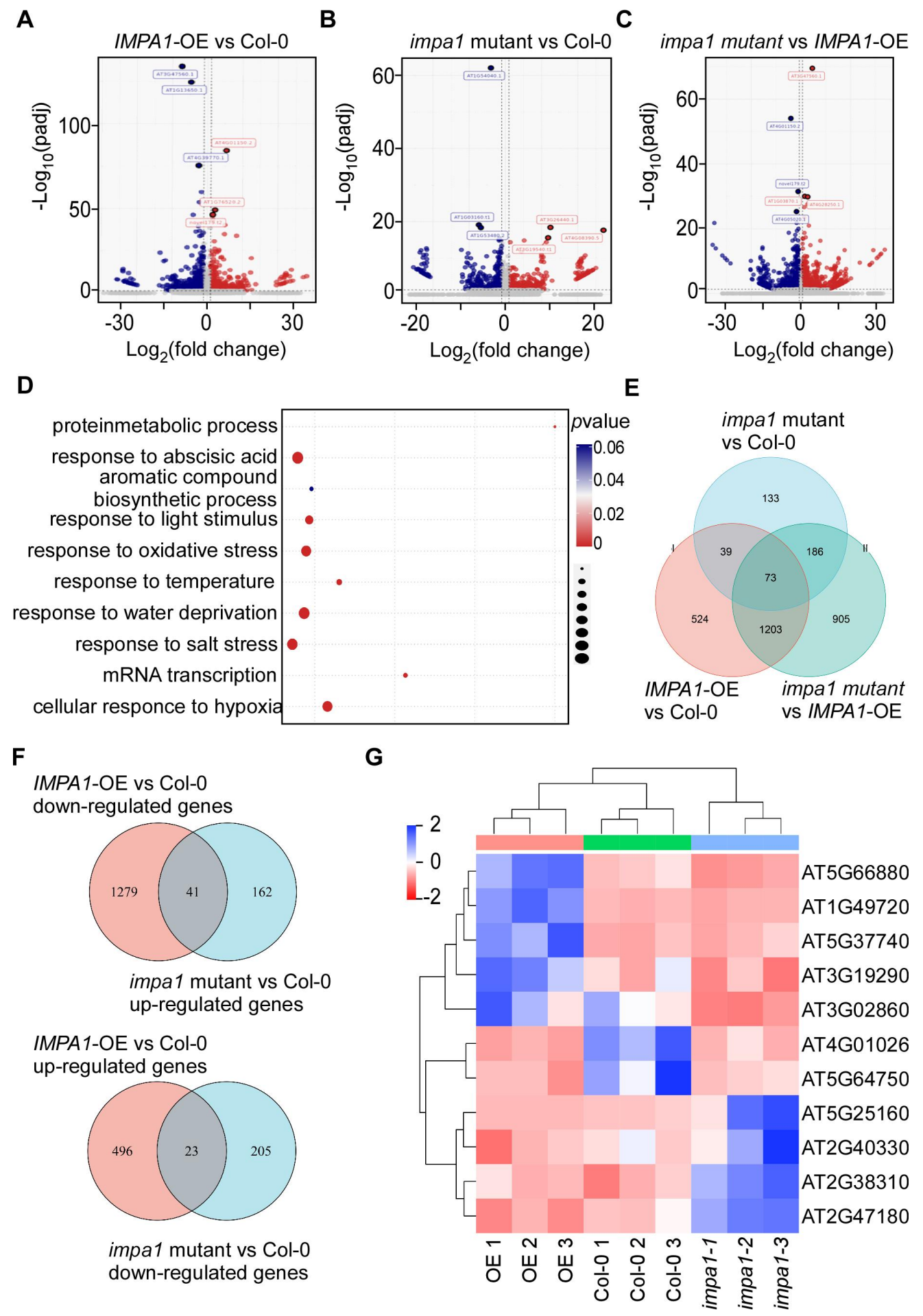

Figure S4. Transcriptome profiling of Col-0, *impa1-1* mutant, and *AtIMPA1*-overexpression lines. (A-C) Volcano plots show differentially expressed genes (DEGs) in *IMPA1*-OE vs. Col-0, *impa1-1* mutant vs. Col-0, and *impa1-1* vs. *IMPA1*-OE. Blue dots represent downregulated genes; red dots represent upregulated genes. (D) GO enrichment analysis of DEGs across all three comparisons. Dot size indicates gene number; color intensity represents *P*-value. (E) Venn diagram shows the overlap of DEGs among the three pairwise comparisons. (F) Venn diagrams show the overlap of upregulated and downregulated genes between *IMPA1*-OE vs. Col-0 and *impa1-1* mutant vs. Col-0. (G) Heatmap displays expression patterns for well-documented ABA-related genes across all genotypes.

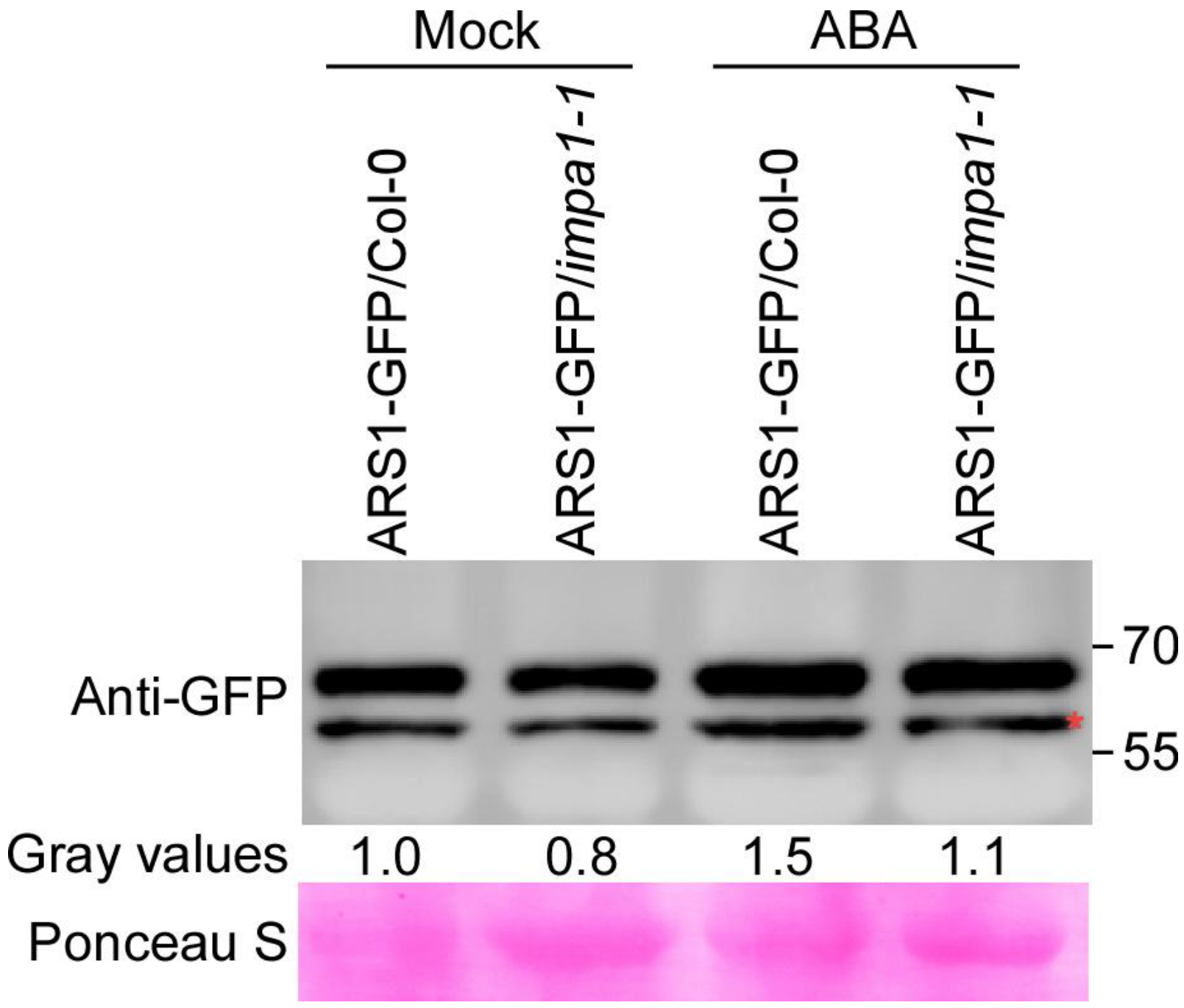

Figure S5. AtARS1 protein levels in Col-0 and the *impa1-1* mutant following ABA treatment. 5-week-old plants were sprayed with 10 μM ABA. Total proteins were extracted from leaf tissues 2 hours post-treatment and subjected to Western blot analysis to detect the accumulation of AtARS1. The asterisk indicates the target strip.

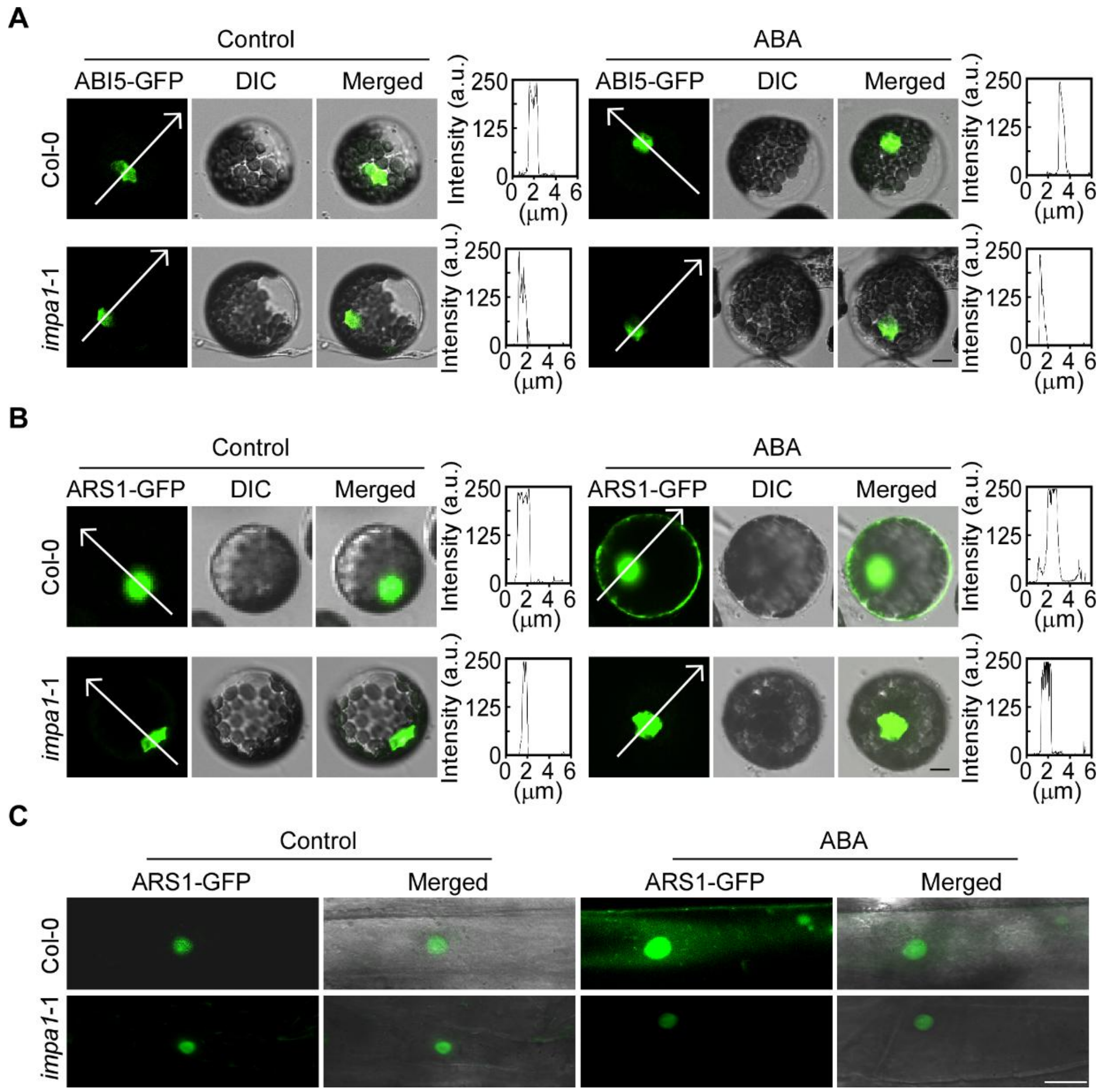

Figure S6. AtIMPA1 is involved in nuclear trafficking of AtARS1 under ABA treatment. (A) Localization of ABI5-GFP in Col-0 and *impa1-1* mutant mesophyll protoplasts under normal and 100 µM ABA treatment. Insets highlight areas analyzed by line scan. The black line represents ABI5-GFP. Scale bar = 50 µm. (B) Localization of ARS1-GFP in Col-0 and *impa1-1* mutant mesophyll protoplasts under normal and 100 µM ABA treatment. Insets highlight areas analyzed by line scan. The black line represents ARS1-GFP. Scale bar = 50 µm. (C) Localization of ARS1-GFP in Col-0 and *impa1-1* mutant roots under normal and 100 µM ABA treatment. Scale bar = 50 µm.

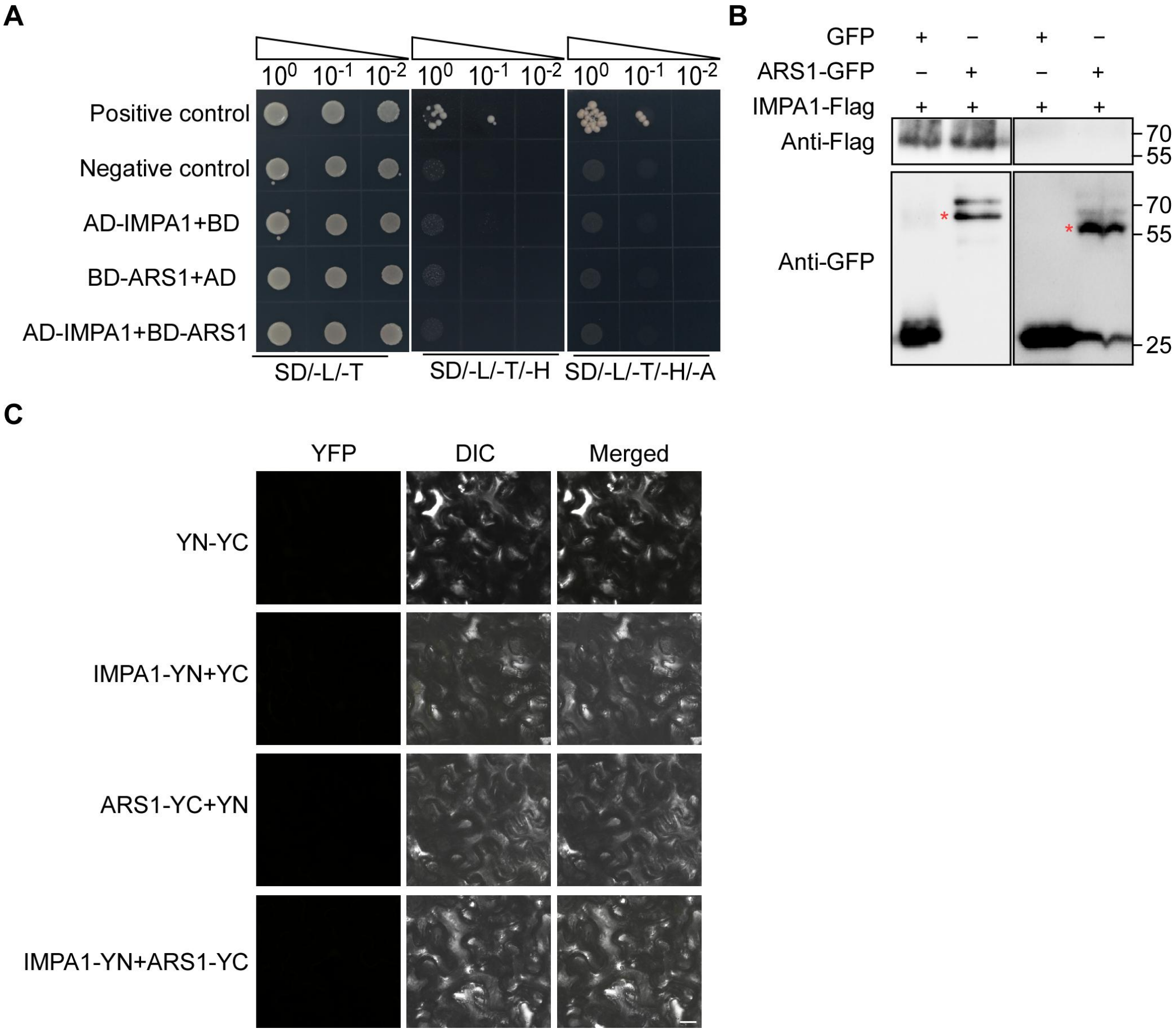

Figure S7. AtIMPA1 does not interact with AtARS1. (A) Yeast two-hybrid (Y2H) assay showing no interaction between AtIMPA1 and AtARS1. The AD-AtIMPA1 and BD-AtARS1 constructs were co-transformed into yeast cells, and the transformants were selected on synthetic defined (SD) medium lacking leucine, tryptophan, histidine, and adenine (SD/-Leu/-Trp/-His/-Ade). (B) Co-immunoprecipitation (Co-IP) assay. AtARS1-GFP was immunoprecipitated with anti-GFP antibodies. Co-immunoprecipitated AtIMPA1-Flag was not detected using anti-Flag antibodies. 35S:GFP was used as a negative control in the assay. (C) Bimolecular fluorescence complementation (BiFC) assay confirming the lack of interaction between AtIMPA1 and AtARS1 in vivo. AtIMPA1 and AtARS1 fused with the split YFP fragments were co-expressed in *N. benthamiana* leaves.

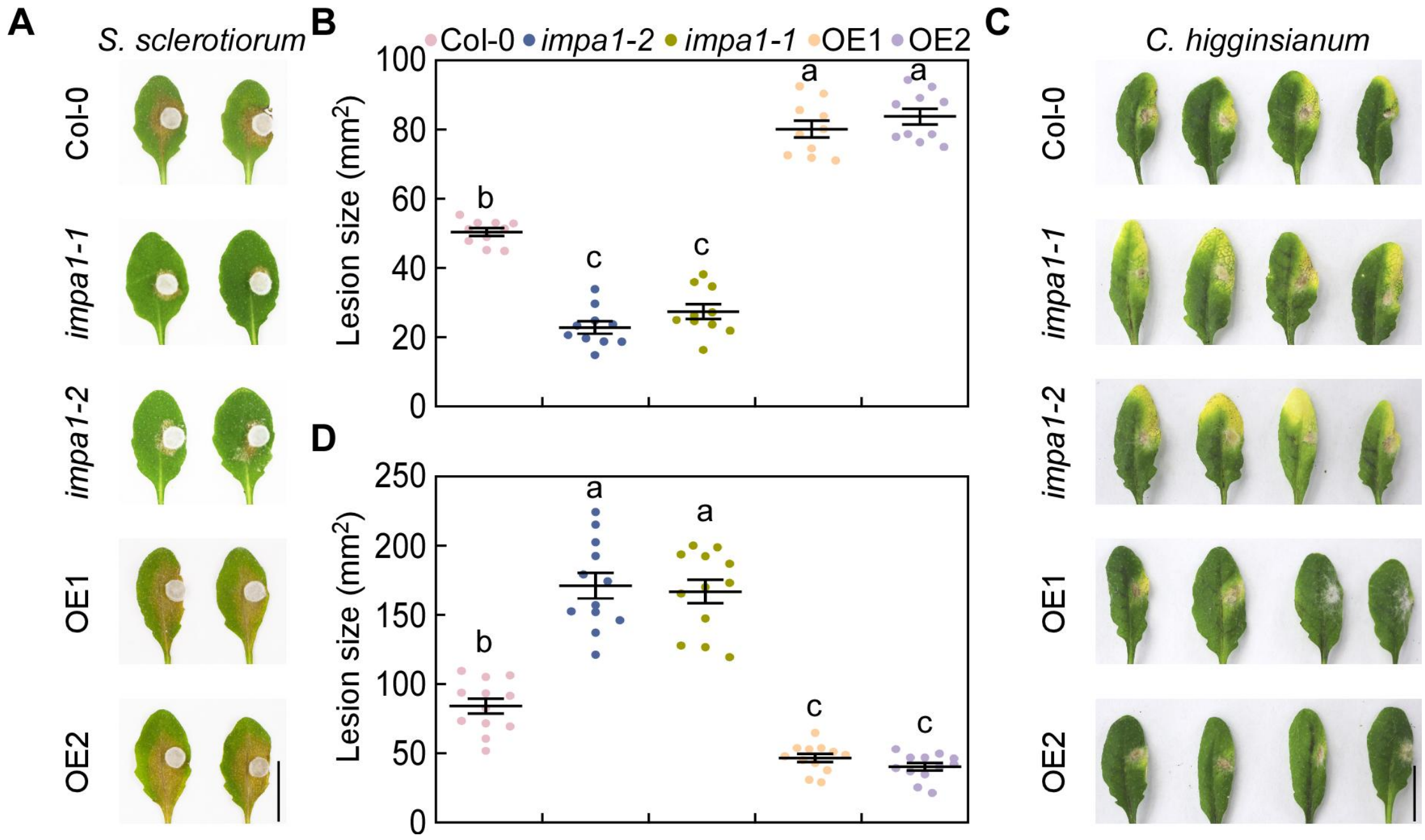

Figure S8. AtIMPA1 modulates resistance to *S. sclerotiorum* and *C. higginsianum*.

(A) Disease symptoms on leaves after infection with *S. sclerotiorum* in Col-0, *impa1-1*, *impa1-2*, OE1 and OE2 plants. Leaves of 5-week-old plants were inoculated with *S. sclerotiorum* at 24 hours. (B) Quantitative analysis of lesion size was performed from 10 leaves fields using ImageJ. Values represent average ± SEM. Statistical analysis was performed by one-way ANOVA with Tukey HSD using GraphPad Prism 8.0.1, and letters represents significant differences at $P<0.01$. Scale bar = 1cm. (C) Disease symptoms on leaves after infection with *C. higginsianum* in Col-0, *impa1-1*, *impa1-2*, OE1 and OE2 plants. Leaves of 5-week-old plants were inoculated with *C. higginsianum* at 3 days. (D) Quantitative analysis of lesion size was performed from 12 leaves fields using ImageJ. Values represent average ± SEM. Statistical analysis was performed by one-way ANOVA with Tukey HSD using GraphPad Prism 8.0.1, and letters represents significant differences at $P<0.01$. Scale bar = 1cm.
